# Burden of antimicrobial resistance in culture-confirmed *Salmonella* Typhi isolates in India from 1977 to 2024: A systematic review and meta-analysis

**DOI:** 10.1371/journal.pntd.0014206

**Published:** 2026-04-16

**Authors:** Vijayalaxmi V. Mogasale, Peixuan Zhang, Jacob John, Habib Hasan Farooqui, Arindam Ray, Vittal Mogasale, Christopher M. Parry, Bhim Gopal Dhoubhadel, W. John Edmunds, Andrew Clark, Kaja Abbas

**Affiliations:** 1 Department of Infectious Disease Epidemiology and Dynamics, London School of Hygiene & Tropical Medicine, London, United Kingdom; 2 School of Tropical Medicine and Global Health, Nagasaki University, Nagasaki, Japan; 3 Institute of Tropical Medicine, Nagasaki University, Nagasaki, Japan; 4 Department of Infectious Disease Epidemiology and International Health, London School of Hygiene & Tropical Medicine, London, United Kingdom; 5 Department of Community Health, Christian Medical College, Vellore, India; 6 College of Medicine, Qatar University, Doha, Qatar; 7 Department of Infectious Disease & Vaccine Delivery, Gates Foundation, New Delhi, India; 8 Graduate School of Public Health, Yonsei University, Seoul, Republic of Korea; 9 Centre for Tropical Medicine and Global Health, University of Oxford, Oxford, United Kingdom; 10 Department of Health Services Research and Policy, London School of Hygiene & Tropical Medicine, London, United Kingdom; 11 Public Health Foundation of India, New Delhi, India; 12 National Institute of Infectious Diseases, Japan Institute for Health Security, Tokyo, Japan; Trinity Medical Science University, School of Medicine, SAINT VINCENT AND THE GRENADINES

## Abstract

**Background:**

Antimicrobial-resistant (AMR) *Salmonella* Typhi (*S*. Typhi) is a persistent public health threat in India. We conducted a systematic review and meta-analysis to estimate the prevalence and temporal trends of AMR *S*. Typhi across Indian states to inform prevention and treatment strategies.

**Methods:**

We systematically reviewed antimicrobial resistance in *S.* Typhi isolates from India, including studies published up to March 31, 2025, with no start-date restriction. We screened eligible articles from PubMed-MEDLINE, EMBASE, Scopus, Web of Science, and AMR surveillance networks and excluded studies on chronic carriers and travellers. We assessed risk of bias using the Risk Of Bias In Non-randomised Studies – of Exposures (ROBINS-E) tool. We conducted meta-analysis using a random-effects model to estimate the prevalence of multidrug resistance (MDR; resistance to chloramphenicol, ampicillin, and co-trimoxazole) and resistance to fluoroquinolones, third-generation cephalosporins, and azithromycin across five-year periods, Indian states, and age groups (<18 versus all ages).

**Results:**

We analysed data from 188 of 4,430 identified studies. MDR declined from 65% (95% CI 63–67) in 1990–94 to nearly 0% (95% CI 0–1) ≥2020. Fluoroquinolone resistance rose from 2% (95% CI 1–2) in 1990–94–85% (95% CI 84–86) in 2015–19, then declined slightly, with similar trends in children but marked variation across Indian states. Resistance to third-generation cephalosporins, azithromycin, and carbapenems remained below 5%, 8%, and 2%, respectively. Our meta-analysis of studies with samples collected from 2015 onwards showed a pooled summary resistance estimate of 82% (95% CI 74–87) for fluoroquinolones, 3% (95% CI 2–4) for third-generation cephalosporins, and 3% (95% CI 1–6) for azithromycin.

**Conclusion:**

Fluoroquinolone resistance in *S*. Typhi in India is high but is declining, while resistance to third-generation cephalosporins and azithromycin is low. As many isolates originated from major cities, caution is warranted when generalising nationally. Continued AMR surveillance is crucial to guide vaccination strategies and treatment decisions.

## Author summary

Our systematic review, based on comprehensive selection criteria, includes a meta-analysis and incorporates newer antibiotics not assessed in earlier reviews from India. We provide prevalence and temporal trends of AMR *S*. Typhi by age and state in India, using data from the earliest available records from 1977 to 2024. Multidrug-resistant (MDR) *S*. Typhi has steadily declined to near zero since 2020. Fluoroquinolone resistance peaked at 85% (95% CI 84–86) between 2015 and 2019 but has since declined. Trends were consistent between children and all-age groups but varied geographically. Resistance to third-generation cephalosporins, azithromycin, and carbapenems remained below 5%, 8%, and 2%, respectively, across three decades. Our findings supports the implementation of typhoid conjugate vaccines (TCVs) and guides typhoid treatment in India. MDR typhoid prevalence is nearly null, while fluoroquinolone resistance remains high but declining. Antimicrobials with low resistance, such as third-generation cephalosporins and azithromycin, remain suitable for typhoid treatment. As AMR trends in children mirror those across all ages, all-age AMR data can be used reliably to guide vaccination decisions for children. The fluctuations in AMR trends highlight the need for proactive AMR surveillance and AMR stewardship. These measures, along with the timely rollout of TCVs, help protect the population against typhoid fever.

## Introduction

Antimicrobial resistance (AMR) is recognised among the top ten global health threats by the World Health Organisation (WHO) [1]. AMR affects our ability to effectively treat infections, resulting in longer illness durations, increased complications, higher mortality rates, and escalating healthcare costs [2]. The lack of new antimicrobials in the pipeline to combat emerging drug-resistant infections makes AMR an unprecedented challenge to global health, rendering the inclusion of AMR under the Global Health Security Agenda [2,3]. *Salmonella enterica* serovar Typhi (*S*. Typhi), the bacterial pathogen which causes typhoid fever, is considered a high-priority pathogen due to AMR [4].

The global burden of disease studies on typhoid fever have estimated that in 2017, there were 10.9 (9.3–12.6) million annual cases and 117,000 (65,000–188,000) annual deaths worldwide, which by 2021 reduced to 9.3 (7.3–11.9) million cases and 107,500 (56,100–180,000) deaths [5,6]. Children have the relatively highest incidence of typhoid fever, with the peak number of cases observed in the 5–9 year age group and declining thereafter [5]. India accounts for about a third of the global burden of typhoid fever, and also sees a similar trend among children [7]. A multi-site, nationally representative typhoid fever surveillance study conducted between 2017 and 2020 estimated an incidence rate of 576–1173 cases per 100,000 person-years [8]. *S*. Typhi is known to develop resistance to multiple antimicrobials, and the WHO recognises it as an increasing priority pathogen [9]. In the updated WHO Bacterial Priority Pathogens List for 2024, *S*. Typhi moved up to 8th place from 18th place in 2017 [4].

The rising AMR in *S.* Typhi is a significant concern [10]. Historically, chloramphenicol has been used to treat typhoid fever since its introduction in the late 1940s, with reports of drug resistance emerging as early as 1962 [11]. Subsequently, the use of ampicillin and co-trimoxazole (trimethoprim-sulfamethoxazole) was promoted, and together, these three drugs are historically considered classical first-line drugs for treating typhoid fever [12]. The term “multidrug-resistant” (MDR) *S.* Typhi was coined to describe the simultaneous resistance to these three first-line drugs [12]. MDR *S*. Typhi was first identified in India during the 1970s. Subsequently, the MDR strain associated with haplotype 58 (H58) became dominant in Southern Asia and spread to Africa on multiple occasions [13]. As MDR *S*. Typhi became widespread in the 1990s, clinicians gradually resorted to other effective antimicrobials, such as fluoroquinolones, as the treatment of choice [12]. However, as the use of fluoroquinolones expanded through the 1990s and 2000s, resistance to these drugs also increased. Consequently, third-generation cephalosporins and azithromycin progressively became preferred antibiotics [12]. In 2016, a typhoid fever outbreak in Pakistan involving *S.* Typhi resistant to third-generation cephalosporins (3GCR) in addition to first-line antimicrobials and fluoroquinolones resulted in the identification of extensively drug-resistant (XDR) strains, posing a public health threat and prompting the introduction of the typhoid conjugate vaccine (TCV) into routine immunisation programmes [10,14]. Current treatment options for XDR *S*. Typhi include azithromycin and broad-spectrum antimicrobials like carbapenems, which require significant healthcare resources [15]. Isolated 3GCR has been reported in India, raising concerns about the emergence of XDR strains [16]. Recent reports of azithromycin resistance (AZR) in India and other nations are of concern, especially since no new antimicrobials are expected in the near future for treating *S*. Typhi infections [17,18].

To support ongoing decision-making regarding TCV, we conducted a study to identify India-specific research priorities by analysing evidence gaps alongside stakeholders’ perceived importance [19,20]. One of the key research priorities identified was the monitoring of AMR in *S*. Typhi. The last systematic review of AMR *S*. Typhi in India summarised the evidence until 2015, and it was necessary to estimate more recent trends [21]. To address this evidence gap, we conducted a systematic review and meta-analysis to estimate the proportion of AMR *S.* Typhi in India. In this review, we expanded on previous systematic reviews of *S*. Typhi AMR [21,22]. We examined nearly five decades (1977–2024) of data to provide the first comprehensive assessment of long-term antimicrobial resistance trends in India, with estimates pooled using meta-analysis. We also included azithromycin and carbapenem resistance and conducted a focused analysis of children to deliver policy-relevant insights to inform treatment guidelines and TCV decision-making.

## Methods

### Ethical approval

This study was approved by the London School of Hygiene & Tropical Medicine (LSHTM) Research Ethics Committee (Ref. No 31427). Informed consent is not applicable as no human subjects were involved.

### Search strategy and selection criteria

We conducted a systematic review of published literature from India, adhering to the PRISMA 2020 guidelines and including all studies available until March 31, 2025, without a restriction on the starting date [23]. We registered the protocol for this review with PROSPERO, the International Prospective Register of Systematic Reviews (CRD42022378201). The initial search covered four databases: PubMed-MEDLINE, EMBASE, Scopus, and Web of Science, using specifically designed search terms (S1A Annex) and did not impose any language restrictions or filters. The key search terms included (typhoid) OR (s typhi) OR (salmonella typhi) OR (typhoidal salmonella) OR (“enteric fever”) AND (resistan*) OR (sensitiv*) OR (suscept*) OR (AMR) AND (india) OR (indian). Additionally, we performed web searches on the Cochrane Library, Google Scholar, reference lists of eligible studies, previous global reviews, and data published by the Antimicrobial Resistance Surveillance and Research Network (AMRSN) under the Indian Council of Medical Research (ICMR), as well as data from the National Programme on AMR Containment by the National Centre for Disease Control (NCDC) to identify eligible studies.

We included studies that reported quantifiable results of antimicrobial susceptibility testing (AST) for *S*. Typhi isolates confirmed from any culture source in acute infections (e.g., blood, bone marrow, cerebrospinal fluid, urine) within the Indian population. We excluded studies on chronic carriers, known AMR cases, travellers, and unknown geographic locations or time periods. Our review focused on studies presenting any of the four groups of AMRs: MDR, fluoroquinolone resistance (FQR), 3GCR, and AZR. Definitions of four groups of AMR and more detailed inclusion and exclusion criteria are available in S1B Annex.

We searched relevant databases, removed duplicate entries, and screened articles using selection criteria at the title, abstract, and full-text review stages. The search involved two researchers independently executing the strategy, followed by cross-verification and discussion to reach consensus.

### Risk of bias assessment

We addressed the risk of bias (RoB) at three levels. First, we applied predefined inclusion and exclusion criteria to ensure that only high-quality studies were included. Second, as major bias is expected in the interpretation of AST, we used clear criteria for standardisation. Finally, we utilised two standard tools to assess RoB: Quality In Prognosis Studies (QUIPS) and Risk Of Bias In Non-randomised Studies - of Exposure (ROBINS E) tools and presented results using Robvis visualisation tool [24,25]. Publication bias was assessed visually using a funnel plot and statistically by Egger’s regression test. The detailed methodology for RoB is available in S2A Annex.

### Data synthesis and analysis

We extracted variables such as study time period, geographical location, study design, settings, age group, culture sources and AST methods. The number of isolates tested for AST under individual drugs and drug groups/categories served as the denominator, while the AST results were used as the numerator to estimate AMR prevalence. Detailed descriptions of the variables and data synthesis are available in S2B Annex.

We created spot maps for each study using the Geographic Information System (GIS) coordinates of the study sites. We organised data into five-year groups (< 1990, 1990–1994,..., 2015–2019, ≥ 2020) and calculated the mean AMR prevalence for the respective drug groups/categories to estimate trends over time using Microsoft Excel. We also assessed trends in AMR prevalence among children and compared them with all-age trends. Additionally, we created a heat map showing AMR prevalence by Indian states, organised into year groups (≤2000, 2001–2010, 2011–2020, > 2020) using the Everviz visualisation tool. Finally, we conducted a meta-analysis to estimate pooled prevalence for each AMR group by combining individual studies using a random-effects model (Stata 18 (StataCorp, 2023) and R Core Team, 2025). We performed two separate meta-analyses: one organised all studies into five-year groups, while the other focused on studies published after 2015, arranged by year, reflecting current antimicrobial susceptibility interpretation standards and recent antimicrobial prescribing practices. We assessed heterogeneity through subgroup analyses by year, visually using forest plots and quantitatively using the I² statistic and its corresponding p-value. As part of our review, we also included a descriptive analysis of other drugs (e.g., carbapenems) identified in the studies.

## Results

**Fig 1:**
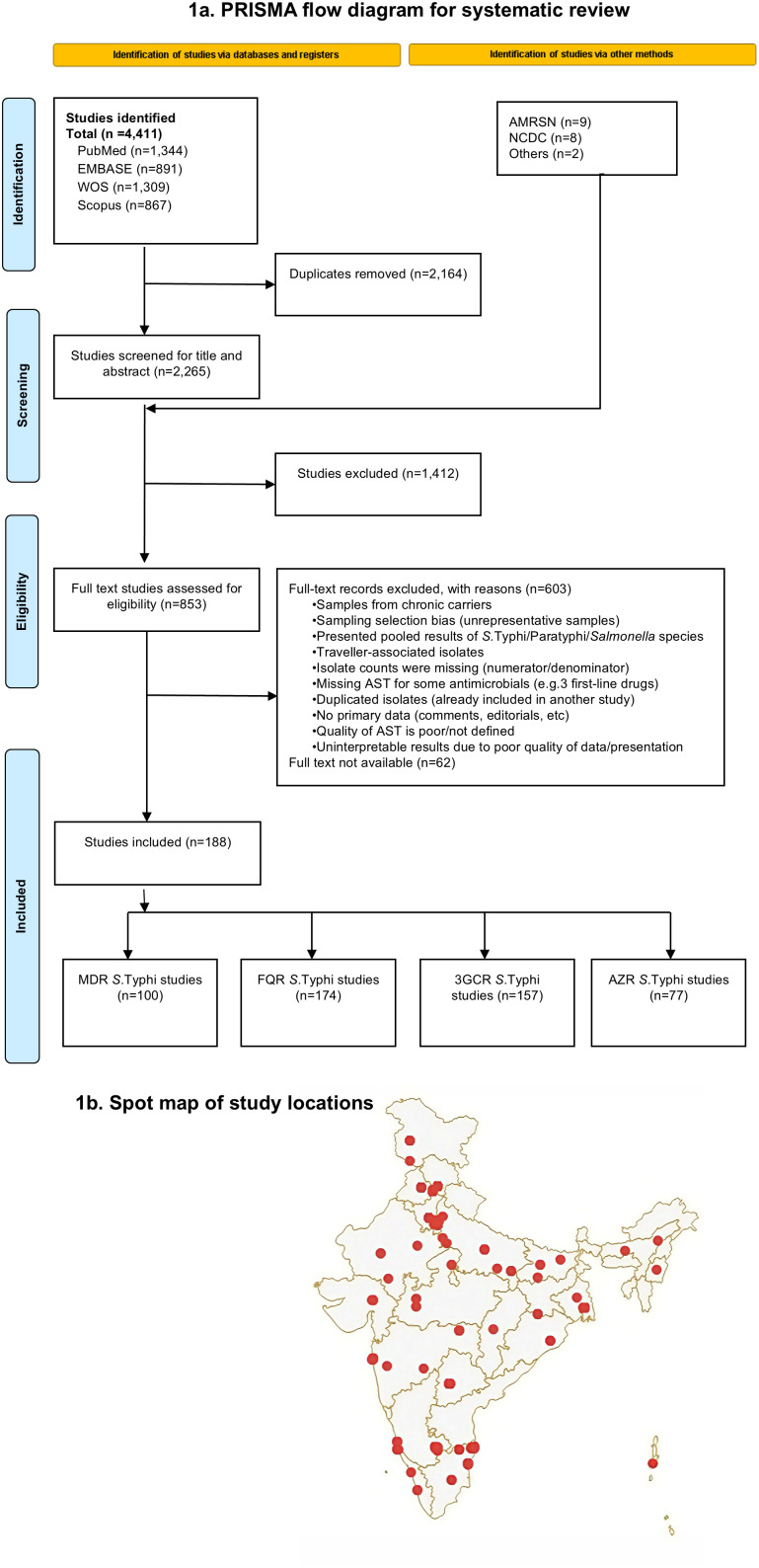
PRISMA flowchart and locations of studies used in assessing the prevalence of antimicrobial resistance (AMR) *Salmonella* Typhi from 1977-2024 in India. a. Preferred Reporting Items for Systematic reviews and Meta-Analyses (PRISMA) flow diagram for systematic review. b. Geographic Information System (GIS) spot map of study locations showing data points from 188 studies. (Base map: India administrative boundaries from Natural Earth (Admin 1 – States and Provinces; https://www.naturalearthdata.com), public domain, rendered using Everviz.).

We identified 4,411 papers from four databases and 19 articles from other sources (AMRSN and NCDC). After removing duplicates and screening titles and abstracts, we included 853 studies for a full-text review, as described in Fig 1a of the Preferred Reporting Items for Systematic reviews and Meta-Analyses (PRISMA) flow chart. Following the full-text review, various exclusions (S2 Annex) led us to include 171 papers from four databases and 17 reports from other sources, resulting in 188 papers for data extraction (S3 Annex). The two RoB tools indicated a low risk of bias for most studies and a moderate risk for others; however, none of the studies were classified as having a high risk of bias overall, with moderate ratings reflecting AST methodological limitations rather than systematic bias (S4 Annex). The characteristics of AST and the source of samples used in these 188 papers are available in S5 Annex.

We analysed 33,651 *S.* Typhi isolates in 188 studies from 1977 to 2024, covering 23 of India’s 36 states and union territories and 315 study data points (Fig 1 b, S5 Annex). We geo-linked 22,765 *S*. Typhi isolates to particular states, while 10,886 isolates couldn’t be geo-linked, details of which are available in S5 Annex. The highest numbers of isolates came from the states/union territories of Delhi (n = 5,891), Karnataka (n = 4,089), West Bengal (n = 2,042), Tamil Nadu (n = 1,549), Maharashtra (1,518), Odisha (1,189) and Punjab (n = 1,119), with a larger volume of samples obtained from major cities of Delhi, Kolkata, Mumbai, Chennai, and Chandigarh (S6 Annex).

There were 100 papers and 176 data points that tested 15,626 isolates for MDR, 174 papers and 286 data points that tested 30,568 isolates for FQR, 157 papers and 257 data points that tested 26,691 isolates for 3GCR, and 77 papers and 138 data points that tested 14,893 isolates for AZR.

**Fig 2:**
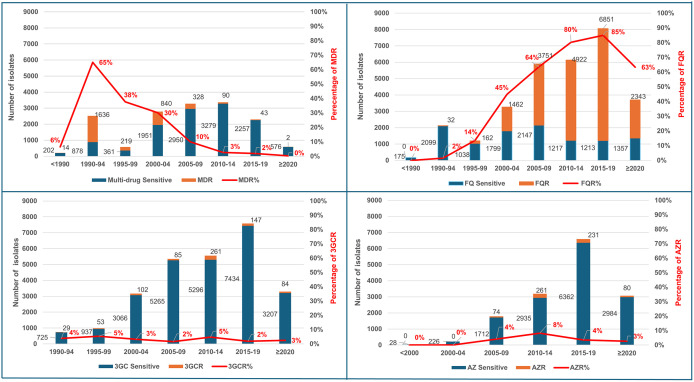
Time trend graph showing prevalence of drug resistance in *Salmonella* Typhi isolates from 1977 to 2024 by 5-year intervals in India. (3GCR = third-generation cephalosporin resistance; AZR = azithromycin resistance; FQR = fluoroquinolone resistance; MDR = multidrug resistance).

**Fig 3:**
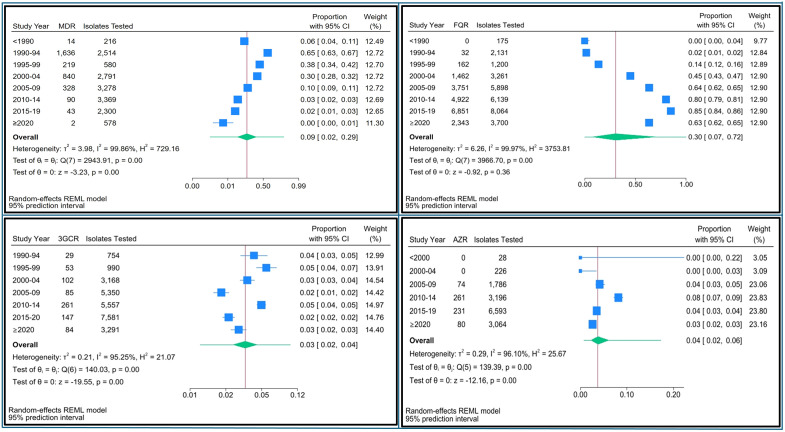
Forest plot showing pooled prevalence of drug resistance in *Salmonella* Typhi isolates from 1977 to 2024 by 5-year intervals in India. (3GCR = third-generation cephalosporin resistance; AZR = azithromycin resistance; FQR = fluoroquinolone resistance; MDR = multidrug resistance).

From 1977 to 2024, 3,172 (20%) of *S*. Typhi isolates were identified as MDR, with a pooled prevalence of 11% (95% CI [8–14]) (S7 Annex). Both time trend analysis and 5-year-groupwise pooled meta-analysis showed that the prevalence of MDR peaked in 1990–94, reaching 78% (95% CI [62–88]) in 1991, and has gradually decreased over the past 30 years, declining to near zero in 2022 (95% CI [0–8]) (Figs 2 and 3, S7 Annex).

**Fig 4:**
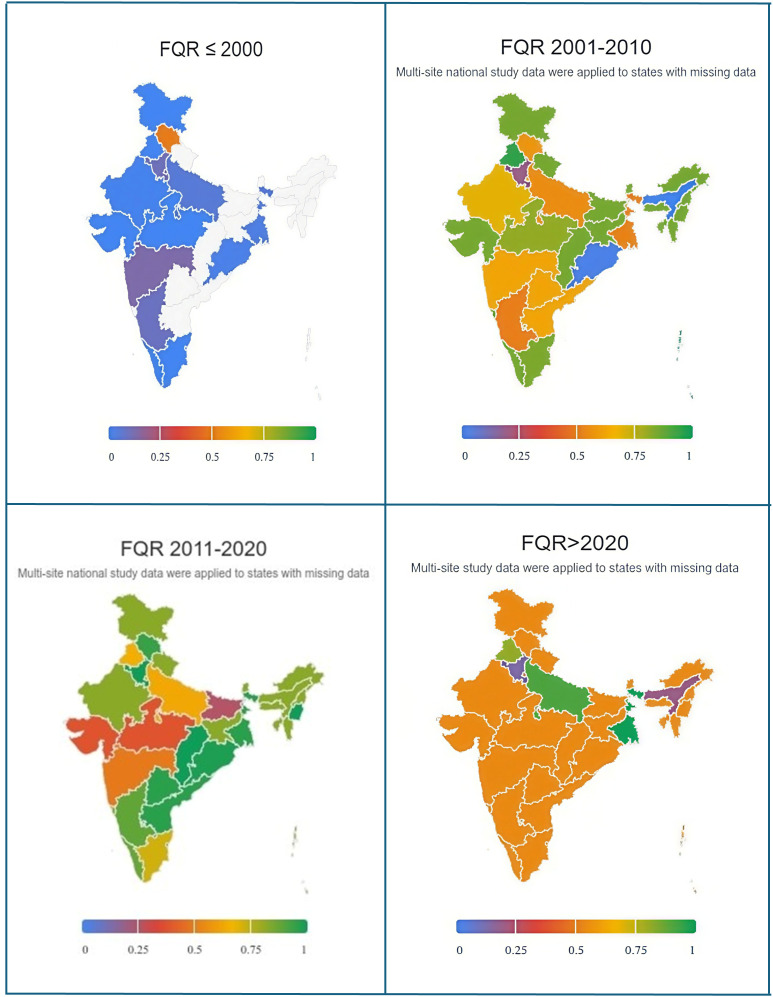
Prevalence of fluoroquinolone resistance (FQR) *Salmonella* Typhi isolates from 1989 to 2024 by 10-year intervals and by Indian states. The colour spectrum indicates prevalence percentages ranging from 1% to 100%. For states without state-specific data, values reflect national multi-site estimates to ensure consistent spatial representation and are not state-level prevalence. (Base map: India administrative boundaries from Natural Earth (Admin 1 – States and Provinces; https://www.naturalearthdata.com), public domain, rendered using Everviz.).

**Fig 5:**
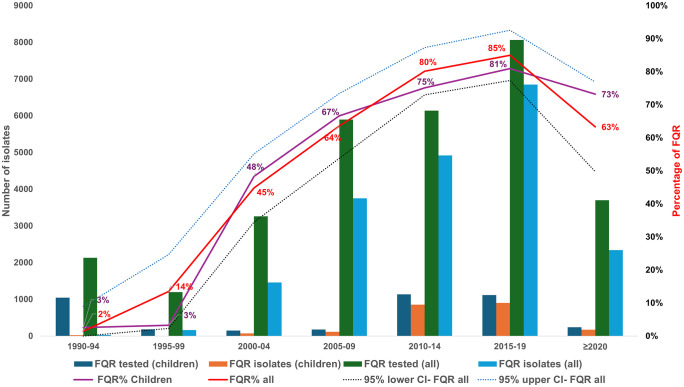
Prevalence of fluoroquinolone resistance (FQR) *Salmonella* Typhi isolates from 1989 to 2024 in children versus all age groups by 5-year intervals in India.

From 1989 to 2024, 19,553 (64%) of *S*. Typhi isolates were FQR-positive, with a pooled prevalence of 58% (95% CI [50–65]) (S8 Annex). The prevalence of FQR has risen steadily since the late 1990s, peaking between 2015 and 2019 at 85% (95% CI [84–86]), with the highest point reaching 94% (95% CI [80–98]) in 2017 (Figs 2 and 3, S8 Annex). However, FQR prevalence declined thereafter. The FQR prevalence varied widely across Indian states and over time, with the highest rates observed in South Indian states, West Bengal, and populous Northern states such as Uttar Pradesh, as shown in the prevalence heat map (Fig 4). About 13% (4,044/30,568) of all *S*. Typhi isolates tested for FQR were collected exclusively from children (<18 years old). Among these, 2,155 (53%) isolates were FQR, showing similar prevalence trends to the all-age FQR data across the years (Fig 5).

The pooled prevalence of 3GCR was 3% (95% CI [2–4]; n = 761) and remained below 5% over the last 30 years (Figs 2 and 3). Similarly, the pooled prevalence of AZR was 4% (95% CI [1–6]; n = 646) and remained below 8% from 1988 through 2024 (Figs 2 and 3).

Among 3,052 samples tested from 1993 to 2023, 1.6% (n = 49) exhibited resistance to the carbapenem class of beta-lactam antibiotics (meropenem, imipenem). These results are heavily influenced by three studies that reported high carbapenem resistance and, therefore, require cautious interpretation. On the other hand, more recent studies conducted after 2020 tested 1,675 isolates and found a much lower carbapenem resistance rate of only 0.4% (n = 6).

In the meta-analysis, funnel plots of all four AMR groups showed visual asymmetry except for FQR (S9 Annex) and Egger’s test demonstrated significant small study effects except for FQR (MDR- β = -3.57, p = < 0.001; FQR- β = -0.55, p = 0.252; 3GCR- β = -2.71, p = < 0.001; AZR- β = -2.80, p = < 0.001). Consequently, individual studies with a sample size of five or fewer were excluded from the meta-analysis. Using a random effects model, the pooled prevalence of FQR *S*. Typhi among studies that collected samples after 2015 was found to be 82% (95% CI [74–87]) (S10 Annex). In contrast, the pooled prevalence for MDR, 3GCR, and AZR remained below 3% (S11-S13 Annexes). All four meta-analyses exhibited high heterogeneity, with I² values exceeding 73% and p-values <0.0001.

## Discussion

Our systematic review and meta-analysis provide the most updated evidence on AMR *S*. Typhi in India. The MDR *S.* Typhi peaked at 65% (95% CI 63–67) in 1990–1994 and has declined to nearly zero since 2020. The FQR peaked at 85% (95% CI 84–86) in 2015–19 but has since declined, with similar patterns noted in children, though varied by state. Resistance to third-generation cephalosporins, azithromycin, and carbapenems remained stable in all five-year periods over the last 30 years, staying below 5%, 8%, and 2%, respectively, in all time periods.

A previous review on AMR *S*. Typhi in India reported increasing FQR from 2001 to 2015 [21]. Our systematic review shows this increase continued until 2015–19, followed by a slight decline thereafter. Earlier Indian and global reviews [21,22] reported a decrease in MDR *S*. Typhi until 2015, and our analysis confirms that this downward trend persisted through 2023. While a previous Indian review noted rising 3GCR until 2015 [21], our findings indicate resistance has remained consistently below 5%. We also investigated resistance patterns to azithromycin and carbapenems, which had not been previously reported. AZR remained below 8%, while carbapenem resistance was negligible throughout. The global review has similarly reported a decline in MDR, accompanied by an increase in FQR, which aligns with our results [22]. However, besides our data, insights into the trends beyond 2020 are limited.

These changes in resistance patterns parallel the treatment practices for typhoid in India. Clinicians commonly used first-line antimicrobials against *S.* Typhi in the 1980s; MDR emerged in the 1990s. As a result, clinicians switched to fluoroquinolones, leading to an increase in FQRs and a decline in MDR, as shown in our trends [10,26]. These decreasing in MDR and increasing in FQR trends were also documented in site-specific studies. A longitudinal study tracking AMR in *S*. Typhi from 2000 to 2015 in three major hospitals in India showed a similar trend of declining MDR and increasing FQR [9]. When FQR peaked in the 2010s, clinicians again shifted to other antimicrobials, such as cephalosporins, which reflected in declining FQR trends after 2020. Available evidence on typhoid fever treatment practices in India supports the theory that changes in prescription patterns may be linked to changes in AMR (S14 Annex). A cross-sectional analysis of medical audit data on antimicrobial prescriptions for typhoid fever from 2013 to 2015 revealed a decrease in the proportion of quinolone prescriptions, alongside an increase in cephalosporin prescriptions [27].

Currently, 3GCR, AZR, and carbapenem resistance remain low. However, any increase in 3GCR in the context of high levels of FQR along with MDR could lead to the emergence of extensively drug-resistant *S*. Typhi [28]. Therefore, ongoing monitoring of AMR is crucial. Phylogeographical studies have identified multiple waves of international and intercontinental transmission of AMR *S*. Typhi from Asia, particularly from India, followed by local spread and replacement of susceptible strains, further highlighting the importance of monitoring and controlling AMR in *S*. Typhi [13].

The state-level AMR patterns identified in our study offer critical insights for decision-making on vaccine implementation. In 2022, India’s National Technical Advisory Group on Immunisation recommended introducing TCVs into the Universal Immunisation Programme [29]. However, the finalisation of vaccination strategies and their implementation are pending. The AMR patterns showed significant variation across states, suggesting that states with the highest AMR prevalence could be prioritised for vaccination efforts. Additionally, we found that the prevalence trend of AMR in children reflects the all-age AMR patterns. Therefore, in the absence of specific age-group data for children, AMR trends in the general population may serve as a valuable proxy for informing vaccination strategies for children.

A major concern with rising AMR is its impact on increasing mortality and morbidity [2], leading to more hospitalisations, longer hospital stays, loss of health-related quality of life, and increased costs. This incremental burden can be substantial in countries with a high prevalence of resistance. A cohort study conducted in eight hospitals across India estimated that treating infections caused by AMR pathogens, including *S*. Typhi, cost 1.8 times higher (US$199 versus US$109) than treating susceptible cases in government hospitals [30]. Additionally, the length of hospital stay for patients with AMR infections was greater, 14 days versus 12 days in the retrospective analysis, and 24 versus 12 days in the prospective study [30]. Estimating the incremental disease and economic burden of AMR *S.* Typhi is crucial for evaluating the importance of typhoid fever control measures. To effectively address the burden of AMR, continued AMR surveillance and comprehensive control strategies involving a multi-sectoral approach, such as antimicrobial stewardship and vaccination, are necessary [31].

Our study has limitations. First, many isolates originated from prominent research sites in New Delhi, Chennai, Kolkata, and Pondicherry, resulting in greater representation. As blood culture and AST are more frequently performed in tertiary centres, these data are likely to over-represent hospitalised or more severe cases. However, we did include several multi-site studies with large sample sizes, which partially mitigated this bias. The data also included some of India’s largest states, providing reasonable national representation. Second, ascertaining the quality of AST was challenging. Researchers reported ASTs at various time periods, and guidelines evolved concurrently; however, researchers may not have kept pace with the changes, and some did not report their quality assurance methods. The lack of clear definitions for AST in earlier years, coupled with changing breakpoints for fluoroquinolones, complicates our ability to determine the reliability of trends in FQR throughout the study period. Similarly, the absence of azithromycin breakpoints before 2015, combined with technical difficulties in interpreting azithromycin AST, makes it difficult to draw definitive conclusions about trends. Third, some studies did not report “intermediately resistant” FQR *S.* Typhi isolates, potentially underestimating prevalence. Fourth, pooled national reports may have overlapped with site-specific studies, causing possible duplication. To minimise duplicate counting, we additionally screened for overlap by author, study site and period; we prioritised studies that were comprehensive and of higher quality. Finally, antimicrobial susceptibility does not always correlate with clinical efficacy, a critical consideration for guidelines.

In conclusion, our systematic review showed that the FQR *S*. Typhi has peaked and is starting to decline. Meanwhile, MDR, 3GCR, and AZR remained low. Given that *S*. Typhi is listed as a high-priority pathogen by the WHO and has the potential to spread internationally, it is crucial to continuously monitor *S*. Typhi AMR in India. It is vital to have timely and appropriate responses, such as prioritising the implementation of the TCV.

## Supporting information

S1 Annex A. Search terms used in four databases on 31 March 2025 (search date 4 April 2025). B. Definition of antimicrobial resistance (AMR) and detailed description of inclusion and exclusion criteria used in the search. (DOCX)

S2 Annex A. Methodology of risk of bias assessment. B. Description of variables used and details of data synthesis. C. Reasons for paper exclusions. (DOCX)

S3 Annex Description of studies included in the systematic review. (DOCX)

S4 Annex Risk of bias assessment shown in six domains using the robvis tool. (TIF)

S5 Annex The characteristics of antimicrobial susceptibility tests (AST), source of samples used, study data points and studies that could not be geo-linked. (DOCX)

S6 Annex Bubble map of 22,765 *S*.Typhi isolates by Indian states identified in the systematic review. Darkness of colour and bubble size are proportional to sample size. (Base map: India administrative boundaries from Natural Earth (Admin 1 – States and Provinces; https://www.naturalearthdata.com), public domain, rendered using Everviz.).
(DOCX)

S7 Annex Forest plot showing proportion of multidrug-resistant (MDR) *S*.Typhi in India by year. (PDF)

S8 Annex Forest plot showing proportion of fluoroquinolone-resistant (FQR) *S*.Typhi in India by year. (PDF)

S9 Annex The funnel plots of four antimicrobial resistance groups showing publication bias. (PDF)

S10 Annex Forest plot showing proportion of fluoroquinolone-resistant (FQR) *S*.Typhi in India since 2015. (PDF)

S11 Annex Forest plot showing proportion of multidrug-resistant (MDR) *S*.Typhi in India since 2015. (PDF)

S12 Annex Forest plot showing proportion of 3^rd^ generation cephalosporin-resistant (3GCR) *S*.Typhi in India since 2015. (PDF)

S13 Annex Forest plot showing proportion of azithromycin-resistant (AZR) *S*.Typhi in India since 2015. (PDF)

S14 Annex Prescription practices and their potential implications for AMR. (DOCX)

S15 Annex Variables extracted and study-wise data used in the systematic review. (XLSX)

S16 Annex PRISMA 2020 for abstract checklist indicating where each reporting item is addressed in the abstract. (DOCX)

S17 Annex PRISMA 2020 checklist indicating where each reporting item is addressed in the manuscript. (DOCX)
