# Supplementary material for: Burden of antimicrobial resistance in culture-confirmed Salmonella Typhi isolates in India from 1977 to 2024: A systematic review and meta-analysis
Source: PLoS Negl Trop Dis. 2026 Apr 16;20(4):e0014206. doi: 10.1371/journal.pntd.0014206 (PMC13108858; doi:10.1371/journal.pntd.0014206)
Supplement: S1 Annex — (DOCX) [file pntd.0014206.s001.docx]

**Annex 1a:** Search terms used in four databases on 31 March 2025 (search date 4 April 2025) **Database 1: PubMed (All Fields)**

|  | **Concept 1** | **Concept 2** | **Concept 3** | **Final** |
| --- | --- | --- | --- | --- |
| **Key concepts** | **Typhoid** | **Antimicrobial Resistance** | **India** |  |
| Key words/search terms | (typhoid) OR (s typhi) OR (salmonella typhi) OR (typhoidal salmonella) OR ("enteric fever") | (resistan*) OR (sensitiv*) OR (suscept*) OR (AMR) | (india) OR (indian) | (Concept 1) AND  (Concept 2) AND  (Concept 3) |
| Total results | 26,982 | 3,757,097 | 1,048,058 | **1,344** |

**PubMed Search string**

Search: ((typhoid) OR (s typhi) OR (salmonella typhi) OR (typhoidal salmonella) OR ("enteric fever")) AND ((resistan*) OR (sensitiv*) OR (suscept*) OR (AMR)) AND ((india) OR (indian))) Filters: from 1000/1/1 - 2025/3/31 Sort by: Most Recent

(("typhoid fever"[MeSH Terms] OR ("typhoid"[All Fields] AND "fever"[All Fields]) OR "typhoid fever"[All Fields] OR "typhoid"[All Fields] OR "typhoidal"[All Fields] OR ("salmonella typhi"[MeSH Terms] OR ("salmonella"[All Fields] AND "typhi"[All Fields]) OR "salmonella typhi"[All Fields] OR "s typhi"[All Fields]) OR ("salmonella typhi"[MeSH Terms] OR ("salmonella"[All Fields] AND "typhi"[All Fields]) OR "salmonella typhi"[All Fields]) OR (("typhoid fever"[MeSH Terms] OR ("typhoid"[All Fields] AND "fever"[All Fields]) OR "typhoid fever"[All Fields] OR "typhoid"[All Fields] OR "typhoidal"[All Fields]) AND ("salmonella"[MeSH Terms] OR "salmonella"[All Fields] OR "salmonellas"[All Fields] OR "salmonella s"[All Fields] OR "salmonellae"[All Fields])) OR "enteric fever"[All Fields]) AND ("resistan*"[All Fields] OR "sensitiv*"[All Fields] OR "suscept*"[All Fields] OR ("appl magn reson"[Journal] OR "altern med rev"[Journal] OR "amr"[All Fields])) AND ("india"[MeSH Terms] OR "india"[All Fields] OR "india s"[All Fields] OR "indias"[All Fields] OR ("indian"[All Fields] OR "indian s"[All Fields] OR "indians"[All Fields]))) AND (1000/1/1:2025/3/31[pdat])

**Database 2: EMBASE (mp.)**

|  | **Concept 1** | **Concept 2** | **Concept 3** | **Final** |
| --- | --- | --- | --- | --- |
| **Key concepts** | **Typhoid** | **Antimicrobial Resistance** | **India** |  |
| Key words/search terms | typhoid.mp. or s typhi.mp. or salmonella typhi.mp. or typhoidal salmonella.mp. or enteric fever.mp. | resistan*.mp. or sensitiv*.mp. or suscept*.mp. or AMR.mp. | india.mp. or indian.mp. | (Concept 1) AND  (Concept 2) AND  (Concept 3) |
| Total results | 37,407 | 5,131,882 | 435,889 | **891** |

**Database 3: Web of Science (All Fields)**

|  | **Concept 1** | **Concept 2** | **Concept 3** | **Final** |
| --- | --- | --- | --- | --- |
| **Key concepts** | **Typhoid** | **Antimicrobial Resistance** | **India** |  |
| Key words/search terms | typhoid (All Fields) or s typhi (All Fields) or "salmonella typhi"(All Fields) or "typhoidal salmonella" (All Fields) or "enteric fever"(All Fields) | resistan* (All Fields) or sensitiv* (All Fields) or suscept* (All Fields) or AMR (All Fields) | india (All Fields) or indian (All Fields) | (Concept 1) AND  (Concept 2) AND  (Concept 3) |
| Total results | 16,987 | 6,329,192 | 3,079,324 | **1,309** |

**Database 4: Scopus (TITLE-ABS-KEY)**

|  | **Concept 1** | **Concept 2** | **Concept 3** | **Final** |
| --- | --- | --- | --- | --- |
| **Key concepts** | **Typhoid** | **Antimicrobial Resistance** | **India** |  |
| Key words/search terms | (TITLE-ABS-KEY (typhoid) OR TITLE-ABS-KEY ( {s typhi}/ ) OR TITLE-ABS-KEY ( {salmonella typhi} ) OR TITLE-ABS-KEY ({typhoidal salmonella}) OR TITLE-ABS-KEY ( {enteric fever} ) ) | (TITLE-ABS-KEY (resistan*) OR TITLE-ABS-KEY (sensitiv*) OR TITLE-ABS-KEY (suscept*) OR TITLE-ABS-KEY ( amr ) ) | (TITLE-ABS-KEY (india) OR TITLE-ABS-KEY ( indian ) ) | (Concept 1) AND  (Concept 2) AND  (Concept 3) |
| Total results | 39,185 | 8,035,053 | 932,520 | **867** |

**Annex 1b:** Definition of antimicrobial resistance (AMR) and detailed description of inclusion and exclusion criteria used in the search

We defined MDR as concurrent resistance to chloramphenicol, ampicillin/amoxicillin, and co-trimoxazole. FQR was defined as resistance to ciprofloxacin, ofloxacin, nalidixic acid, or pefloxacin. Since the definition of ciprofloxacin resistance has been revised several times, we accepted the definitions used by the respective studies during their reporting periods, in accordance with the Clinical and Laboratory Standards Institute (CLSI) guidelines at the time. Resistance to ceftriaxone or other drugs in the same class was categorised as 3GCR. The azithromycin resistance was defined based on CLSI breakpoints (susceptible MIC ≤ 16 μg/mL and resistant MICs ≥ 32 μg/mL), first established in 2015 (1). For studies before 2015, we accepted the AZR definitions used in the paper if they were based on the then-available standards. We considered the actual reported value when studies reported AST results using either disc diffusion (DD) or minimum inhibitory concentration (MIC) methods or a combination of both. If there was any discrepancy, we used the lower of the two values. We considered AMR in *S*. Typhi isolates when they were reported as "resistant," "non-susceptible," "intermediately resistant," or "intermediately susceptible" based on DD or MIC values, following CLSI guidelines, the National Committee for Clinical Laboratory Standards (NCCLS), or other standards, as indicative of AMR.

We included all studies that reported quantifiable antimicrobial susceptibility test (AST) results for *S*. Typhi isolates, confirmed by any culture source or study type (prospective, retrospective, hospital-based, community-based, or laboratory-based).

We excluded studies that isolated *S*. Typhi from healthy individuals or chronic carriers, such as samples collected more than one year after the onset of acute typhoid fever or repeated samples of stool, urine, bile, or duodenal string cultures. Additionally, studies that selected only *S.* Typhi isolates with known AMR status were excluded. We also excluded studies involving travel-associated cases unless the origin was documented within India. Furthermore, studies that did not report both the geographical location and the sample collection years were excluded. Other exclusions were studies that did not provide results for any of the four antimicrobial groups included in this study, those that presented pooled results spanning more than 5 years, or studies that combined S. Typhi with S. Paratyphi and/or other Salmonella species' susceptibility data.

**References**

1. CLSI. Clinical and Laboratory Standards Institute Performance Standards for Antimicrobial Susceptibility Testing; 25th Informational Supplement. CLSI Document M100-S25, Clinical and Laboratory Standards Institute, Wayne, PA. 2015.
