# Supplementary material for: Burden of antimicrobial resistance in culture-confirmed Salmonella Typhi isolates in India from 1977 to 2024: A systematic review and meta-analysis
Source: PLoS Negl Trop Dis. 2026 Apr 16;20(4):e0014206. doi: 10.1371/journal.pntd.0014206 (PMC13108858; doi:10.1371/journal.pntd.0014206)
Supplement: S2 Annex — (DOCX) [file pntd.0014206.s002.docx]

**Annex 2a: Methodology of risk of bias assessment**

We anticipated significant bias in the conduct of AST and in the interpretation of its findings. To ensure standardisation, we established clear criteria for categorising isolates based on DD and/or MIC values. We also verified adherence to CLSI, NCCLS, and other standard guidelines, as well as internal quality control measures. The QUIPS assesses RoB in six domains, of which only four were relevant to our study (risk of bias in the study population, risk of bias in the measurement of AMR, risk of bias in the study attrition, and risk of bias due to statistical analysis and reporting) (1). This tool was also used in previous systematic reviews that classified bias as low, moderate, or high (2). The ROBINS-E tool also employs defined domains for assessing RoB; only six were relevant to our study and were used in our analysis, including RoB due to confounding, measurement of AMR, sample selection, AST methodology, missing data, and reporting (3) . Studies were then classified into four categories: low, some, high, and very high RoB. We intended to exclude studies with high or very high RoB and, in the end, presented the results of the RoB analysis, combined across six domains, using a visualisation tool called Robvis (3).

**Annex 2b: Description of variables used and details of data synthesis**

Our extracted data included variables such as the author and publication year with DOI, study year and study period, geographical location (including state, city name, and GIS coordinates), study design (prospective or retrospective), study setting (hospital, community, laboratory, or outbreak), age groups, culture source, type of AST (DD/MIC), the standards (CLSI/ NCCLS /other) used with internal quality control measures, and culture-confirmed *S*. Typhi isolates and isolates tested for AST. We created a spot map of all identified studies conducted in India, using GIS coordinates to pinpoint the study locations using the Everviz visualisation tool (Base map: India administrative boundaries from Natural Earth (Admin 1 – States and Provinces; https://www.naturalearthdata.com), public domain, rendered using Everviz).

The number of isolates tested for AST under individual drugs and drug groups/categories served as the denominator, while the AST results were used as the numerator. This allowed us to calculate the prevalence of AMR. The prevalence of FQR and 3GCR was estimated based on the highest resistance among respective individual drugs under that category (e.g. FQR was estimated based on the highest resistance for ciprofloxacin, ofloxacin, nalidixic acid, or pefloxacin in the same isolate). If a study reported only susceptible cases, we reverse-calculated the number of resistant cases using the total number of isolates tested and the reported susceptibility estimates. Similarly, if a study reported the number of isolates tested and the percentage of susceptibility or resistance, we reverse-calculated the number of resistant cases.

We assigned study years based on the data collection year. If data collection spanned two years, we selected the year with the longer data collection duration. When the durations were equal, we chose the latter year. For studies in which data collection spanned more than two years, we utilised the median year of data collection. If the median year overlapped with two data-collection years, we used the latter year's median. If the year of data collection was not specified in the publication, we assigned it as one year earlier than the publication year.

**Annex 2C**: **Reasons for study exclusions**

The main reasons for excluding full-text papers were: samples from chronic carriers, sampling selection bias (unrepresentative samples), missing susceptibility results or tested isolate counts (numerator/denominator), absence of AST data for one of the first-line antimicrobials, additional duplication (already included in another study by same author group), traveller-associated isolates, lack of primary data (e.g., comments, editorials), combined results for *S*. Typhi/Paratyphi/other *Salmonella* spp., pooled results over more than five years, poor or undefined quality of AST, unpredictable data due to poor presentation, and unavailability of full texts.

**References**

1. Hayden JA, van der Windt DA, Cartwright JL, Côté P, Bombardier C. Assessing bias in studies of prognostic factors. Ann Intern Med. 2013;158(4):280–6.
2. Britto CD, John J, Verghese VP, Pollard AJ. A systematic review of antimicrobial resistance of typhoidal *Salmonella* in India. Indian J Med Res. 2019;149(2):151–63.
3. Higgins JPT, Morgan RL, Rooney AA, Taylor KW, Thayer KA, Silva RA, et al. A tool to assess risk of bias in non-randomized follow-up studies of exposure effects (ROBINS-E). Environ Int. 2024;186:108602.
