# Supplementary material for: Burden of antimicrobial resistance in culture-confirmed Salmonella Typhi isolates in India from 1977 to 2024: A systematic review and meta-analysis
Source: PLoS Negl Trop Dis. 2026 Apr 16;20(4):e0014206. doi: 10.1371/journal.pntd.0014206 (PMC13108858; doi:10.1371/journal.pntd.0014206)
Supplement: S3 Annex — (DOCX) [file pntd.0014206.s003.docx]

**Annex 3: Studies included in the systematic review**

| Study no | First Author | Publication Year | Indian States/UT | Study place Name | Setting | Study design | Study year | Culture Source | AST (Antibiotic Sensitivity Test) | Test standard | | Identification (DOI/PMICD) |
| --- | --- | --- | --- | --- | --- | --- | --- | --- | --- | --- | --- | --- |
| 1 | Rao RS | 1981 | Tamil Nadu | Chennai | Hospital | NA | 1977 | Blood & Bone Marrow | MIC | NA | | 10.1016/0035-9203(81)90008-0 |
| 2 | Anand AC | 1990 | West Bengal | Kolkata | Outbreak | NA | 1989 | Blood | Stokes disc diffusion method | NA | | 10.1016/0140-6736(90)90635-I |
| 3 | Jesudasan M | 1990 | Tamil Nadu | Vellore | Hospital | NA | 1990 | NA | NA | NA | | 10.1016/0140-6736(90)91783-7 |
| 4 | Ayyagari A | 1991 | Chandigarh | Chandigarh | Outbreak-Hospital based report | ?Prospective | 1990 | Blood | NA | NA | | 10.1016/0035-9203(91)90066-8 |
| 5 | Koul PB | 1991 | New Delhi | New Delhi | Hospital | Prospective | 1990 | Blood & Bone Marrow | Stokes disc diffusion method | NA | | Accession Number: 1752652 |
| 6 | Sen S | 1991 | New Delhi | New Delhi | Hospital | ?Prospective | 1990 | Blood | Disc diffusion | NA | | PMCID: 1752663 |
| 7 | Arora RK | 1992 | West Bengal | Kolkata | Outbreak | Prospective | 1990 | Blood & Bone Marrow | Stokes disc diffusion method | NA | | PMCID: 1601498 |
| 8 | Dar L | 1992 | New Delhi | New Delhi | Hospital | NA | 1990 | Blood | Kirby-Bauer disk diffusion | NA | | 10.1007/BF02759988 |
| 9 | Mehta A | 1992 | Maharashtra | Mumbai | Hospital | NA | 1990 | Blood | Kirby-Bauer disk diffusion | NCCLS | | 10.1001/jama.1992.03480120052032 |
| 10 | Mishra S | 1992 | New Delhi | New Delhi | Hospital | ?Prospective | 1990 | Blood | Stokes disc diffusion method | NA | | PMCID: 1506095 |
| 11 | Ramanan A | 1992 | Karnataka | Bangalore | Hospital | Prospective | 1990 | Blood | Kirby-Bauer disk diffusion | NA | | PMCID: 1601484 |
| 12 | Rao RS | 1992 | Pondicherry | Pondicherry | Outbreak | Prospective | 1990 | Blood&Stool | Kirby-Bauer disk diffusion | NCCLS | | 10.1016/0035-9203%2892%2990573-U |
| 13 | Garg RA | 1993 | New Delhi | New Delhi | Hospital | ?Prospective | 1991 | Blood | NA | NA | | Accession Number: 8132267 |
| 14 | Sharma A | 1993 | Haryana | Rohtak | Hospital | ?Prospective | 1991 | Blood | NA | NA | | Accession Number: 8406706 |
| 15 | Biswal N | 1994 | Pondicherry | Pondicherry | Hospital | Prospective | 1990 | Blood, Bone marrow, urine, stool, CSF | NA | NA | | PMCID: 7890344 |
| 16 | Buch NA | 1994 | Jammu & Kashmir | Srinagar | Hospital | Prospective | 1988 | Blood | NA | NA | | PMCID: 7883352 |
| 17 | Garg K | 1994 | Rajastan | Jaipur | Hospital | ?Prospective | 1991 | Blood | Stokes disc diffusion method | NA | | PMCID: 7875843 |
| 18 | Harish R | 1994 | Jammu & Kashmir | Jammu | Hospital | NA | 1991 | NA | NA | NA | | Accession Number: 7875844 |
| 19 | Sethuraman S | 1994 | Kerala | Kozhikode (Calicut) | Hospital | Prospective | 1992 | Blood | NA | NA | | 10.1080/02724936.1994.11747736 |
| 20 | Agrawal V | 1995 | Maharashtra | Nagpur | Hospital | NA | 1991 | NA | NA | NA | | PMCID: 8919117 |
| 21 | Gidvani CH | 1995 | Uttar Pradesh | Agra | Hospital | NA | 1994 | Blood & Bone marrow | Stokes disc diffusion method | NA | | 10.1016/s0377-1237(17)30934-6 |
| 22 | Rajajee S | 1995 | Tamil Nadu | Chennai | Hospital | NA | 1992 | Blood | Disc diffusion | NA | | 10.1093/tropej/41.1.52 |
| 23 | Rathish KC | 1995 | Karnataka | Bangalore | Hospital | NA | 1992 | Blood | Kirby-Bauer disk diffusion & MIC | NA | | 10.1007/BF02755065 |
| 24 | Takkar VP | 1995 | Punjab | Ludhiana | Hospital | NA | 1991 | Blood | NA | NA | | PMCID: 8613320 |
| 25 | Misra OP | 1996 | Uttar Pradesh | Varanasi | Hospital | NA | NA | Blood | NA | NA | | 10.1093/tropej/42.5.310-a |
| 26 | Verma M | 1996 | Punjab | Ludhiana | Hospital | Retrospective case records | 1992 | Blood | NA | NA | | PMCID: 8772845 |
| 27 | Khosla SN | 1998 | Haryana | Rothak | Hospital | Prospective | 1992 | Blood & Bone Marrow | NA | NA | | 10.1177/004947559802800419 |
| 28 | Shanahan PMA | 1998 | Tamil Nadu | Vellore | Hospital | NA | 1993 | Blood | MIC | NA | | 10.1128/jcm.36.6.1595-1600.1998 |
| 29 | Chitnis V | 1999 | Madhya Pradesh | Indore | Hospital | NA | 1991 | NA | NA | NA | | 10.1016/S0140-6736(05)75549-5 |
| 30 | Rodrigues C | 1999 | Maharashtra | Mumbai | Hospital | NA | 1998 | NA | MIC | NA | | PMCID: 10416332 |
| 31 | Sood S | 1999 | New Delhi | New Delhi | Hospital | Retrospective | 1994 | Blood | Stokes disc diffusion method | BSAC | | 10.1016/s0140-6736(99)00637-6 |
| 32 | Das U | 2000 | Odisha | Rourkela | Hospital | Retrospective | 1998 | Blood | Kirby-Bauer disk diffusion |  | | PMCID: 11217269 |
| 33 | Kabra SK | 2000 | Gujarat | Ahmedabad | Hospital | Prospective | NA | Blood | NA |  | | 10.1177/004947550003000404 |
| 34 | Nath G | 2000 | Uttar Pradesh | Varanasi | Hospital | NA | 1998 | NA | Disc diffusion | NCCLS | | PMCID: 10882709 |
| 35 | Sanghavi SK | 2000 | Maharashtra | Pune | Hospital | NA | 1998 | Blood,  Stool(3),  pus(1) |  |  | Accession Number: 12583416 | |
| 36 | Gautam V | 2002 | Haryana | Rohtak | Hospital | NA | 1997 | Blood | Stokes disc diffusion method | Standard method interpretation | | 10.1590/S1413-86702002000600003 |
| 37 | Mehta M | 2002 | Chandigarh | Chandigarh | Hospital | Retrospective | 2000 | Blood | Stokes disc diffusion method | NA | | 10.1053/jinf.2002.0985 |
| 38 | Kadappu KK | 2003 | Karnataka | Manipal | Hospital | Prospective | 2000 | Blood &Bone marrow | | NA | NA | |
| 39 | Tankhiwale SS | 2003 | Maharashtra | Nagpur | Hospital | NA | 2002 | Blood | NA (but other paratyphi study mentions disc diffusion) | NA | | Accession Number: 37456005 |
| 40 | Madhulika U | 2004 | Pondicherry | Pondicherry | Hospital | NA | 2002 | Blood | Kirby-Bauer disk diffusion | NCCLS | | PMCID: 15347861 |
| 41 | Rupali P | 2004 | Tamil Nadu | Vellore | Hospital | NA | 2000 | Blood | Disc diffusion | NCCLS | | 10.1016/j.diagmicrobio.2003.12.002 |
| 42 | Safdar A | 2004 | Punjab | Ludhiana | Hospital | Prospective | 2000 | Blood, Stool | Disc diffusion | NCCLS | | 10.1159/000077808 |
| 43 | Chowta MN | 2005 | Karnataka | Mangalore | Hospital | Retrospective | 2000 | Blood | NA | NA | | 10.4103/0255-0857.16054 |
| 44 | Dutta S | 2005 | West Bengal | Kolkata | Community | Prospective surveillance | 2004 | Blood | Kirby-Bauer disk diffusion & MIC by E test | NA | | 10.1128/AAC.49.4.1662-1663.2005 |
| 45 | Kadhiravan T | 2005 | New Delhi | New Delhi | Hospital | Prospective | 2002 | Blood | Disc diffusion & MIC by E test | NCCLS 2000 | | 10.1186/1471-2334-5-37 |
| 46 | Misra RN | 2005 | Maharashtra | Mumbai | Outbreak | Prospective | 2000 | Blood & Bone marrow | Stokes disc diffusion method | NA | | 10.1016/s0377-1237(05)80011-5 |
| 47 | Reddy KR | 2005 | Tamil Nadu | Chennai | Hospital | NA | 2002 | Blood & Bone marrow | Disc diffusion | NCCLS | | 10.4103/0255-0857.16601 |
| 48 | Das U | 2006 | Odisha | Rourkela | Hospital | NA | 2003 | Blood | Kirby-Bauer disk diffusion | NCCLS | | PMCID: 16926466 |
| 49 | Lakshmi V | 2006 | Andhra Pradesh | Hyderabad | Hospital | NA | 2002 | Blood | Mini API ID32E strip (France) | NCCLS | | 10.4103/0255-0857.19894 |
| 50 | Manchanda V | 2006 | New Delhi | New Delhi | Hospital | Prospective | 2004 | Blood | Disc diffusion | NCCLS | | 10.4103/0255-0857.25182 |
| 51 | Mohanty S | 2006 | New Delhi | New Delhi | Hospital | Retrospective lab records | 1999 | Blood | Disc diffusion | NCCLS | | 10.1017/s0950268805005844 |
| 52 | Ray P | 2006 | Chandigarh | Chandigarh | Hospital | NA | 2002 | Blood | Disc diffusion & MIC | NCCLS | | WOS:000240574900018 |
| 53 | Walia M | 2006 | New Delhi | New Delhi | Hospital | Retrospective | 2002 | Blood | Disc diffusion | NCCLS | | 10.1016/j.trstmh.2006.02.015 |
| 54 | Banerjee CA | 2007 | Maharashtra | Maharashtra | Outbreak | Prospective | 2003 | Blood | Disc Diffusion & MIC | NA | | 10.1016/s0377-1237(07)80005-0 |
| 55 | Bhatia JK | 2007 | Uttar Pradesh | Mathura | Hospital | NA | 2004 | Blood | NA | NA | | 10.1016/s0377-1237(07)80136-5 |
| 56 | Capoor MR | 2007 | New Delhi | New Delhi | Hospital | NA | 2001 | Blood | Kirby-Bauer Disc diffusion & MIC by agar dilution | CLSI 2006 | | 10.1099/jmm.0.47170-0 |
| 57 | Garg A | 2007 | Uttar Pradesh | Varanasi | Hospital | Retrospective | 2003 | Blood | Kirby-Bauer disk diffusion | NCCLS | | Accession Number: 46953993 |
| 58 | Joshi S | 2007 | Karnataka | Bangalore | Hospital | NA | 2004 | Blood | Disc diffusion |  | | 10.1016/j.trstmh.2006.05.009 |
| 59 | Kumar R | 2007 | New Delhi | New Delhi | Hospital | Prospective clinical trial | 2003 | Blood | NA | NA | | 10.1007/s12098-007-0024-z |
| 60 | Sen B | 2007 | West Bengal | Kolkata | Community | Prospective surveillance | 2004 | Blood | Kirby-Bauer disk diffusion | CLSI | | PMCID: 17642505 |
| 61 | Jog S | 2008 | Maharashtra | Mumbai | Hospital | Retrospective | 2003 | Blood,  Bone  marrow,  stool | Disc diffusion | NA | PMCID: 18702385 | |
| 62 | Kumar S | 2008 | New Delhi | New Delhi | Hospital | Prospective | 1999 | Blood | Kirby-Bauer disk diffusion | CLSI | | 10.1099/jmm.0.2008/001719-0 |
| 63 | Raveendran R | 2008 | New Delhi | New Delhi | Hospital | NA | 2005 | Blood | Kirby-Bauer disk diffusion | CLSI | | 10.4103/0255-0857.38858 |
| 64 | Vidyalakshmi K | 2008 | Karnataka | Mangalore | Hospital | NA | 2006 | Blood | Disc diffusion | CLSI | | 10.1258/td.2007.070271 |
| 65 | Capoor MR | 2009 | New Delhi | New Delhi | Hospital | NA | 2007 | Blood | MIC mainly & Kirby-Bauer disk diffusion | CLSI 2006 | | 10.1099/jmm.0.47853-0 |
| 66 | Gupta S | 2009 | Karnataka | Bangalore | Hospital | Retrospective | 2008 | Blood | NA |  | | 10.1016/s0377-1237(09)80093-2 |
| 67 | Gupta V | 2009 | Punjab | Chandigarh | Hospital | NA | 2006 | Blood | Kirby-Bauer disc diffusion | CLSI | | 10.1258/td.2008.070452 |
| 68 | Arora D | 2010 | Karnataka | Manipal | Hospital | NA | 2002 | Blood &Bone marrow | Kirby-Bauer disk diffusion | NCCLS | | WOS:000278855400011 |
| 69 | Verma S | 2010 | Himachal Pradesh | Shimla | Hospital | Retrospective | 2000 | Blood &Bone marrow | Kirby-Bauer disk diffusion | NA | | 10.4103/0255-0857.58730 |
| 70 | Yashvanth R | 2010 | Karnataka | Mangalore | Hospital | NA | 2006 | Blood | Kirby-Bauer disk diffusion | CLSI | | Accession Number: 359999327 |
| 71 | Aggarwal A | 2011 | New Delhi &Kolkata | New Delhi &Kolkata | Hospital | Prospective clinical trial | 2010 | Blood | Kirby-Bauer disk diffusion | NA | | 10.1007/s13312-011-0093-y |
| 72 | Alam MS | 2011 | New Delhi | New Delhi | Hospital | Prospective | 2009 | Blood | Kirby-Bauer disk diffusion | CLSI | | 10.4103/0975-7406.90106 |
| 73 | Bhattacharya SS | 2011 | Odisha | Rourkela | Hospital | Retrospective | 2007 | Blood | Kirby-Bauer disk diffusion | CLSI | | PMC3103178 |
| 74 | Jain S | 2011 | Uttar Pradesh | Lucknow | Hospital | NA | 2007 | Blood | Disc diffusion & MIC with E test for Azithro | CLSI & BSAC | | 10.1016/S1201-9712%2811%2960180-X |
| 75 | Muthu G | 2011 | Tamil Nadu | Chennai | Hospital | NA | 2008 | Blood, Bone marrow & stool(1) | Disc diffusion | CLSI | | Accession Number: 364051729 |
| 76 | Sathimoorthi T | 2011 | Tamil Nadu | Tiruchirappalli district | Community | Prospective randomised active surveillance | 2009 | Blood & Stool | Disc diffusion | CLSI 2003 | | 10.1007/s10389-011-0414-6 |
| 77 | Holt KE | 2012 | West Bengal | Kolkata | Community | Prospective cluster RCT | 2005 | Blood | Kirby Bauer disc diffusion & MIC | CLSI | | 10.1371/journal.pntd.0001490 |
| 78 | Joshi S | 2012 | AMR Network India | 15 centers | Hospital, lab | Retrospective | 2008 | Blood,few pus, urine, stool | Kirby Bauer disc diffusion & MIC | CLSI | | 10.4103/2224-3151.206930 |
| 79 | Kumar A | 2012 | Karnataka | Manipal | Hospital | Retrospective case records | 2007 | NA | NA | NA | | 10.4103/0970-0218.103475 |
| 80 | Menezes G | 2012 | Pondicherry | Pondicherry | Hospital | NA | 2005 | Blood | Kirby Bauer disc diffusion & MIC for cipro | CLSI (2007) | | 10.1111/j.1469-0691.2011.03546.x |
| 81 | Pathak A | 2012 | Madhya Pradesh | Ujjain | Hospital | Prospective | 2008 | Blood | Kirby Bauer disc diffusion | CLSI | | 10.2147/idr.S30043 |
| 82 | Rai S | 2012 | New Delhi | New Delhi | Hospital | Retrospective HIS system | 2007 | Blood | Disc diffusion & MIC with E test | CLSI+BSAC (Azithro) | | 10.4103/0255-0857.93017 |
| 83 | Sabiha ST | 2012 | Maharashtra | Lathur | Hospital | NA | 2011 | Blood & Stool | Kirby Bauer disc diffusion |  | | 10.3329/bjms.v11i1.9821 |
| 84 | Shetty AK | 2012 | Karnataka | Mangalore | Hospital | Prospective | 2010 | Blood | Kirby Bauer disc diffusion | CLSI | | 10.4103/0974-2727.105585 |
| 85 | Thamizhmani R | 2012 | Andaman & Nicobar Islands | Port Blair | Hospital | Prospective | 2010 | Blood & Stool | Kirby Bauer disc diffusion | CLSI | | PMC3461725 |
| 86 | Choudhary A | 2013 | Tamil Nadu | Chennai | Hospital | NA | 2010 | Blood | Kirby Bauer disc diffusion & MIC E test | CLSI | | PMC3724263 |
| 87 | Garg A | 2013 | Himachal Pradesh | Shimla | Hospital | Prospective | 2012 | Blood | Kirby Bauer disc diffusion & MIC | NA | | 10.4103/0255-0857.115641 |
| 88 | Gupta V | 2013 | Chandigarh | Chandigarh | Hospital | NA | 2008 | Blood | Kirby-Bauer disk diffusion & MIC by agar dilution & E test | CLSI | | PMC3773355 |
| 89 | Jain S | 2013 | New Delhi | New Delhi | Hospital | Retrospective | 2010 | Blood | Phoenix & Kirby-Bauer disk diffusion | CLSI, BSAC (Azithro) | | 10.3855/jidc.3030 |
| 90 | Kumar Y | 2013 | Maharashtra | 14 hospitals | CRI Reference lab | NA | 2009 | Blood | Kirby-Bauer disk diffusion |  | | PMC3799410 |
| 91 | Singla N | 2013 | Chandigarh | Chandigarh | Outbreak | Retrospective | 2010 | Blood | Kirby-Bauer disk diffusion | CLSI 2009 | | 10.1016/S1995-7645(13)60017-6 |
| 92 | Venkatesh BM | 2013 | Karnataka | Bangalore | Hospital | NA | 2012 | Blood | Kirby-Bauer disk diffusion & MIC for Cipro & Azithro | CLSI 2012 | | 10.4103/0377-4929.125418 |
| 93 | Duggal S | 2014 | New Delhi | New Delhi | Hospital | Retrospective lab based | 2012 | Blood | Kirby-Bauer disk diffusion | CLSI | | Accession Number: 372649783 |
| 94 | Dutta S | 2014 | West Bengal | Kolkata | 3 hospitals | Prospective | 2011 | Blood | Kirby Bauer disc diffusion & MIC by E test | CLSI 2013 | | 10.1371/journal.pone.0101347 |
| 95 | Elumalai S | 2014 | Tamil Nadu | Chennai | Hospital& lab | ?Retrospective | 2009 | Blood | Kirby Bauer disc diffusion | CLSI 2011 | | 10.1099/jmm.0.068486-0 |
| 96 | Geetha VK | 2014 | Pondicherry | Pondicherry | Hospital | NA | 2011 | Blood | Kirby Bauer disc diffusion & MIC by Agar dilution for ceftri | CLSI 2012 | | 10.4103/0255-0857.124292 |
| 97 | Mathew R | 2014 | Tamil Nadu | Chennai | Hospital | Prospective | 2011 | Blood | Kirby Bauer disc diffusion | CLSI | | Accession Number: 372649702 |
| 98 | Srirangaraj S | 2014 | Pondicherry | Pondicherry | Hospital | Prospective | 2012 | Blood | Kirby Bauer disc diffusion & MIC with E test | NA | | 10.4066/amj.2014.2010 |
| 99 | AMRSN-Annual report 2014 | 2014 | 5 zones of India | NA | Laboratory | Prospective | 2014 | Blood | Disc diffusion | CLSI | | https://iamrsn.icmr.org.in/index.php/amrsn/amrsn |
| 100 | Misra R | 2015 | Uttar Pradesh | Lucknow | Hospital | NA | 2013 | Blood | Kirby Bauer disc diffusion & E test | CLSI 2014&2015 | | 10.1093/trstmh/trv036 |
| 101 | Uppal B | 2015 | New Delhi | New Delhi | Hospital | Cross-sectional | 2013 | Stool | modified Stokes’ disc diffusion | NA | | 10.7860/jcdr/2015/11965.5619 |
| 102 | Chande CA | 2016 | Maharashtra | Mumbai | Hospital | NA | 2011 | Blood | Kirby Bauer disc diffusion & MIC | NA | | 10.4103/0255-0857.174100 |
| 103 | Gopal M | 2016 | Tamil Nadu | Chennai | Hospital | NA | 2013 | Blood, stool, bone marrow | MIC | CLSI | | 10.7860/jcdr/2016/17677.8153 |
| 104 | Misra R | 2016 | Uttar Pradesh | Lucknow | Hospital | NA | 2014 | Blood | Kirby Bauer disc diffusion & MIC | CLSI 2014 | | 10.1016/j.ijid.2016.02.202 |
| 105 | Ramesh U | 2016 | 18 regions of India (4 zones) | 18 regions of India |  | NA | 2014 | Blood | Disc diffusion | CLSI 2015 | | 10.4103/0255-0857.180393 |
| 106 | Roy JS | 2016 | Assam | Jorhat town | "Community, Outbreak | Prospective | 2014 | Blood | Disc diffusion | CLSI | | 10.4103/0971-5916.200902 |
| 107 | Sania KM | 2016 | Manipur | Imphal | Hospital | Cross-sectional | 2012 | Blood | Kirby Bauer Disk-diffusion | CLSI | | 10.13040/IJPSR.0975-8232.7%289%29.3715-19 |
| 108 | Sharvani R | 2016 | Karnataka | Bangalore | Hospital | Retrospective | 2013 | Blood | Kirby Bauer Disk-diffusion & MIC | CLSI | | 10.7860/jcdr/2016/18102.7753 |
| 109 | Shenoy B | 2016 | Karnataka | Bangalore | Hospital | Retrospective case records | 2008 | Blood | Kirby-Bauer disk diffusion | CLSI | | Conference abstract |
| 110 | Singh CL | 2016 | Uttar Pradesh | Meerut | Hospital | Retrospective | 2013 | Blood | Kirby Bauer Disk-diffusion | CLSI, BSAC (Azithro) | | 10.1016/j.mjafi.2015.07.007 |
| 111 | Vala S | 2016 | Gujarat | Ahmedabad | Hospital | Retrospective | 2011 | Blood | Kirby Bauer Disk-diffusion | CLSI | | 10.1097/MJT.0000000000000094 |
| 112 | AMRSN-Anuual report 2016 | 2016 | 5 zones of India | 20 RC, 1 NC(AIIMS,Delhi):North,South,West,Central,East | Laboratory | Prospective | 2016 | Blood | Disc diffusion | CLSI | | https://iamrsn.icmr.org.in/index.php/amrsn/amrsn |
| 113 | Behl P | 2017 | Chandigarh | Chandigarh | Hospital | NA | 2011 | Blood, pus & stool | Kirby-Bauer disk diffusion | CLSI | | 10.4103/ijmr.IJMR_862_14 |
| 114 | Dahiya S | 2017 | 4 centers | New Delhi,Chandigarh,Vellore,Pondicherry |  | Prospective | 2015 | Blood | Kirby-Bauer disk diffusion | CLSI | | 10.4103/ijmm.IJMM_16_382 |
| 115 | Das S | 2017 | West Bengal | Kolkata | Community & hospital | Prospective&Retrospective | 1999 | Blood | Kirby-Bauer disk diffusion & MIC by E test | CLSI 2014&2015 | | 10.1016/j.ijmm.2016.11.006 |
| 116 | Harichandran D | 2017 | Kerala | Kochi | Hospital | Prospective | 2012 | Blood, urine (1 sample) | Kirby-Bauer disk diffusion & MIC by agar dilution | CLSI | | 10.2147/idr.S126209 |
| 117 | Iyer RN | 2017 | Andhra Pradesh | Hyderabad | Hospital | Retrospective | 2007 | Blood | Disc diffusion & MIC with E test | CLSI 2013 | | 10.1007/s10096-017-3073-x |
| 118 | Patel SR | 2017 | Uttar Pradesh | Varanasi | Hospital | Prospective | 2012 | Blood | Kirby Bauer disc diffusion |  | | 10.7860/jcdr/2017/23330.9973 |
| 119 | Purighalla S | 2017 | Karnataka | Bangalore | Community outbreak | Prospective | 2016 | Blood | MIC-Phoenix | CLSI | | 10.4103/ijmr.IJMR_1201_16 |
| 120 | Ramachandran A | 2017 | Tamil Nadu | Chennai | Hospital | Prospective | 2016 | Blood | Disc diffusion | CLSI | | 10.7860/jcdr/2017/30150.10637 |
| 121 | Swain N | 2017 | Odisha | Bhubaneswar | Hospital | Retrospective | 2014 | Blood | NA | CLSI 2015 | | 10.5958/0976-5506.2017.00095.X |
| 122 | AMRSN-Anuual report 2017 | 2017 | 5 zones of India | 20 RC, 1 NC(AIIMS,Delhi):North,South,West,Central,East | Laboratory | Prospective | 2017 | Blood | Disc diffusion | CLSI | | https://iamrsn.icmr.org.in/index.php/amrsn/amrsn |
| 123 | NCDC(NARS-Net)-Annual Report 2017 | 2017 | 13 state medical colleges | NA | Laboratory | Prospective | 2017 | Blood | Disc diffusion | CLSI | | https://ncdc.mohfw.gov.in/reports/ |
| 124 | Bandyopadhyay R | 2018 | Tamil Nadu | Vellore | Hospital | Retrospective | 2010 | Blood & Bone marrow | Disc diffusion & MIC with E test | NA | | 10.1177/0049475518758884 |
| 125 | Gupta V | 2018 | Chandigarh | Chandigarh | Hospital | ?Prospective | 2017 | Blood | Disc diffusion | CLSI | | 10.4103/0970-258x.262899 |
| 126 | Makkar A | 2018 | New Delhi | New Delhi | Hospital | Ambispective | 2011 | Blood | Kirby-Bauer disc diffusion & MIC for Cipro | CLSI | | 10.14712/18059694.2018.130 |
| 127 | Sharma P | 2018 | New Delhi | New Delhi | Hospital | Retrospective | 2005 | Blood | Disc diffusion & MIC with E test | CLSI 2016 | | 10.4103/ijmm.IJMM_17_412 |
| 128 | Sur D | 2018 | 5 states | Chandigarh, Gurgaon, Kolkata, Vellore, Manipal | Hospital | Retrospective cross-sectional | 2015 | Blood |  | CLSI | | 10.1093/infdis/jiy502 |
| 129 | AMRSN-Anuual report 2018 | 2018 | 5 zones of India | 20 RC, 1 NC(AIIMS,Delhi):North,South,West,Central,East | Laboratory | Prospective | 2018 | Blood | Disc diffusion | CLSI | | https://iamrsn.icmr.org.in/index.php/amrsn/amrsn |
| 130 | NCDC(NARS-Net)-Annual Report 2018 | 2018 | 16 state medical colleges,14 states | NA | Laboratory | Prospective | 2018 | Blood | Disc diffusion | CLSI | | https://ncdc.mohfw.gov.in/reports/ |
| 131 | Chitkara AJ | 2019 | New Delhi | New Delhi | Hospital | Retrospective case records | 2011 | Blood | Vitek 2(automated) | NA | | 10.1007/s13312-019-1686-0 |
| 132 | Chitkara AJ | 2019 | New Delhi | New Delhi | Hospital | Retrospective case records | 2015 | Blood | Vitek 2(automated) | NA | | 10.1007/s13312-019-1686-0 |
| 133 | Das D | 2019 | Chattisgarh | Raipur | Hospital | Retrospective | 2016 | Blood | Kirby-Bauer disk diffusion | CLSI | | 10.4103/jlp.Jlp_154_18 |
| 134 | Iyer V | 2019 | Gujarat | Ahmedabad | Hospital &Lab based | Retrospective AMR surveillance network | 2014 | Blood | Kirby-Bauer disk diffusion | CLSI | | 10.4103/jgid.jgid_149_18 |
| 135 | Joshi S | 2019 | Karnataka | Bangalore | Hospital | Retrospective | 2002 | Blood | Kirby Bauer disc diffusion & MIC by E test | CLSI | | 10.1016/j.mjafi.2018.08.002 |
| 136 | Khan S | 2019 | Kerala | Kochi | Hospital | NA | 2015 | Blood | Kirby Bauer disc diffusion & MIC | CLSI 2015-2017 | | 10.4103/jlp.Jlp_99_18 |
| 137 | Maity S | 2019 | West Bengal | Kolkata | Hospital | Retrospective cross-sectional | 2017 | Blood | Kirby-Bauer disk diffusion | NA | | 10.14260/jemds/2019/441 |
| 138 | Namhata A | 2019 | West Bengal | Kolkata | Hospital | Retrospective | 2018 | Blood | Vitek 2(automated) & MIC in E strip | CLSI 2019 | | 10.14260/jemds/2019/497 |
| 139 | Patil N | 2019 | 4 zones India | East | Lab based | ?Prospective | 2018 | Blood | MIC by E test |  | | 10.2147/idr.S204618 |
| 140 | Pramanik S | 2019 | West Bengal | Burdwan | Hospital | Prospective cross-sectional | 2015 | Blood | Kirby-Bauer disk diffusion | CLSI | | 10.14260/jemds/2019/809 |
| 141 | Sharma P | 2019 | New Delhi | New Delhi | Hospital | ?Retrospective | 2008 | Blood | Disc diffusion | NA | | 10.4103/ijmr.IJMR_1302_17 |
| 142 | AMRSN-Anuual report 2019 | 2019 | 5 zones of India | 20 RC, 1 NC(AIIMS,Delhi):North,South,West,Central,East | Laboratory | Prospective | 2019 | Blood | Disc diffusion | CLSI | | https://iamrsn.icmr.org.in/index.php/amrsn/amrsn |
| 143 | NCDC(NARS-Net)-Annual Report 2019 | 2019 | 29 state medical colleges in 24 states | NA | Laboratory | Prospective | 2019 | Blood | Disc diffusion | CLSI | | https://ncdc.mohfw.gov.in/reports/ |
| 144 | Britto C | 2020 | Karnataka | Bangalore | Hospital | Prospective surveillance | 2017 | Blood | Kirby Bauer disc diffusion & Vitek 2 | CLSI | | 10.1093/jac/dkz435 |
| 145 | Gupta V | 2020 | Chandigarh | Chandigarh | Hospital | NA | 2019 | Blood | Kirby-Bauer disk diffusion | CLSI | | 10.1055/s-0040-1721163 |
| 146 | Keshav K | 2020 | Bihar | Patna | Hospital | Prospective observational | 2019 | Blood | NA | NA | | Accession Number: 2018084263 |
| 147 | Kumar D | 2020 | Bihar | Gaya | Hospital | Prospective observational | 2019 | Blood | NA | NA | | Accession Number: 2027324951 |
| 148 | Malini A | 2020 | Pondicherry | Pondicherry | Hospital | Prospective cross-sectional | 2016 | Blood | Kirby-Bauer disk diffusion | CLSI | | 10.4103/jcls.jcls_17_20 |
| 149 | Munawer A | 2020 | Karnataka | Manipal | Hospital | Prospective | 2017 | Blood | Kirby Bauer disc diffusion & Vitek 2 | CLSI 2017 | | Accession Number: 2004797848 |
| 150 | Pathak A | 2020 | Madhya Pradesh | Ujjain | Hospital | Prospective Cohort | 2016 | Blood | Kirby-Bauer disk diffusion | CLSI 2018 | | 10.1186/s12879-020-4890-6 |
| 151 | Prakash P | 2020 | Bihar | Madhepura | Hospital | Prospective observational | 2019 | Blood | NA | NA | | Accession Number: 2027098408 |
| 152 | Saigal K | 2020 | New Delhi | New Delhi | Hospital | Prospective surveillance | 2019 | Blood | Vitek 2C& E test | NA | | 10.1016/j.ijid.2020.09.424 |
| 153 | Samajpati S | 2020 | West Bengal | Kolkata | Hospital | Prospective | 2016 | Blood | Kirby Bauer disc diffusion | CLSI 2018 | | 10.1016/j.meegid.2020.104478 |
| 154 | AMRSN-Anuual report 2020 | 2020 | 5 zones of India | 20 RC, 1 NC(AIIMS,Delhi):North,South,West,Central,East | Laboratory | Prospective | 2020 | Blood | Disc diffusion | CLSI | | https://iamrsn.icmr.org.in/index.php/amrsn/amrsn |
| 155 | NCDC(NARS-Net)-Annual Report 2020 | 2020 | 29 state medical colleges in 24 states | NA | Laboratory | Prospective | 2020 | Blood | Disc diffusion | CLSI | | https://ncdc.mohfw.gov.in/reports/ |
| 156 | Anuradha S | 2021 | Rajastan | Jodhpur | Hospital | Retrospective | 2016 | Blood | NA | CLSI | | 10.5005/jp-journals-10071-23922 |
| 157 | Behera JR | 2021 | Odisha | Bhubaneswar | Hospital | Retrospective case records | 2018 | Blood | NA | NA | | 10.7759/cureus.12826 |
| 158 | Dzeyie KA | 2021 | Tamil Nadu | Tiruchirappalli city | Outbreak | Prospective | 2018 | Blood | Kirby-Bauer disk diffusion |  | | 10.1016/j.ijregi.2021.09.006 |
| 159 | Krishna D | 2021 | Karnataka | Mangalore | Hospital | ?Prospective | 2017 | Blood | Kirby-Bauer disk diffusion | CLSI | | 10.1089/mdr.2020.0419 |
| 160 | Manohar P | 2021 | Tamil Nadu | Chennai | Lab based | NA | 2016 | Blood, urine, CSF, bile | Disc diffusion | CLSI | | 10.1093/jacamr/dlab015 |
| 161 | Mehta A | 2021 | Madhya Pradesh | Datia | Hospital | Cross-sectional | 2019 | Blood, stool | Kirby-Bauer disk diffusion | CLSI 2018 | | 10.22159/ajpcr.2021.v14i10.42822 |
| 162 | Saigal K | 2021 | New Delhi | New Delhi | Lab based | Prospective surveillance | 2019 | Blood & CSF(1 sample) | Kirby-Bauer disk diffusion | CLSI 2015 | | 10.1093/infdis/jiab430 |
| 163 | Sinha B | 2021 | New Delhi | New Delhi | Community | Prospective active surveillance | 2019 | Blood | Kirby-Bauer disk diffusion | NA | | 10.1093/infdis/jiab046 |
| 164 | Suman S | 2021 | Bihar | Patna | Hospital | Prospective observational | 2020 | Blood | NA | NA | | Accession Number: 2015738141 |
| 165 | Taneja J | 2021 | Haryana | Faridabad | Hospital | Retrospective | 2018 | Blood | Kirby Bauer disc diffusion &Vitek 2 | CLSI | | 10.24321/0019.5138.202101 |
| 166 | Veeraraghavan B | 2021 | 19 centers | 19 centers | 19 centers | Prospective | 2019 | Blood | Disc diffusion | CLSI | | 10.1093/infdis/jiab144 |
| 167 | AMRSN-Anuual report 2021 | 2021 | 5 zones of India | 20 RC, 1 NC(AIIMS,Delhi):North,South,West,Central,East | Laboratory | Prospective | 2021 | Blood | Disc diffusion | CLSI | | https://iamrsn.icmr.org.in/index.php/amrsn/amrsn |
| 168 | NCDC(NARS-Net)-Annual Report 2021 | 2021 | 35 state medical colleges in 25 states | NA | Laboratory | Prospective | 2021 | Blood | Disc diffusion | CLSI | | https://ncdc.mohfw.gov.in/reports/ |
| 169 | Argimón S | 2022 | Maharashtra | Mumbai | Hospital | NA | 2018 | Blood | Vitek 2 | CLSI 2019 | | 10.1093/cid/ciab897 |
| 170 | Bhumbla U | 2022 | Rajastan | Udaipur | Hospital | Retrospective cross-sectional | 2019 | Blood | VITEK 2 | CLSI | | 10.4103/jfmpc.jfmpc_1976_21 |
| 171 | Biswas M | 2022 | West Bengal | Kolkata | Hospital | Retrospective | 2017 | Blood | Kirby Bauer disc diffusion &Vitek 2 | CLSI | | 10.3390/antibiotics11101308 |
| 172 | Borah P | 2022 | Assam | Kamrup | Community | Prospective | 2021 | Stool | Disc diffusion |  | | 10.1007/s11259-022-09900-z |
| 173 | Gupta V | 2022 | 5 zones of India | NA | Laboratory | Prospective | 2023 | Blood | Disc diffusion | CLSI | | https://iamrsn.icmr.org.in/index.php/amrsn/amrsn |
| 174 | NCDC(NARS-Net)-Annual Report 2022 | 2022 | 41 state medical colleges in 31 states/UT | NA | Laboratory | Prospective | 2022 | Blood | Disc diffusion | CLSI | | https://ncdc.mohfw.gov.in/reports/ |
| 175 | Gupta V | 2023 | Punjab | Ludhiana | Hospital | Retrospective | 2018 | Blood | Kirby Bauer disc diffusion &Vitek 2 | CLSI | | 10.24321/0019.5138.202331 |
| 176 | Indrajith S | 2023 | Tamil Nadu | Tiruchirappalli | Hospital | Prospective seroprevalence | 2019 | Blood | Disc diffusion | CLSI | | 10.1007/s00284-023-03343-8 |
| 177 | Kaira SS | 2023 | Uttar Pradesh | Gaziabad | Hospital | Prospective Cross-sectional | 2022 | Blood | Kirby-Bauer disk diffusion | CLSI 2021 | | 10.7860/JCDR/2023/61269.17629 |
| 178 | Nirmal K | 2023 | New Delhi | New Delhi | Hospital | Prospective observational | 2022 | Blood | Kirby-Bauer disk diffusion | NA | | 10.4081/hls.2023.11345 |
| 179 | Patel AH | 2023 | Gujarat | Ahmedabad | Hospital | Prospective Cross-sectional | 2019 | Blood | Kirby-Bauer disk diffusion | CLSI | | 10.5455/njppp.2023.13.03158202305042023 |
| 180 | Siddiqui T | 2023 | Uttar Pradesh | Lucknow | Hospital | Prospective observational | 2020 | Blood | "Phoenix NMIC/ID-55 panels&Kirby–Bauer’s disc diffusion | CLSI 2020 | | 10.1055/s-0043-1772216 |
| 181 | NCDC(NARS-Net)-Annual Report 2023 | 2023 | 37 state medical colleges in 27 states | NA | Laboratory | Prospective | 2023 | Blood | Disc diffusion | CLSI | | https://ncdc.mohfw.gov.in/reports/ |
| 182 | AMRSN-Annual report 2023 | 2023 | 5 zones of India | NA | Laboratory | Prospective | 2023 | Blood | Disc diffusion | CLSI | | https://iamrsn.icmr.org.in/index.php/amrsn/amrsn |
| 183 | Kadambari A | 2023 | Bihar | Patna | Hospital | Prospective | 2018 | Blood | Kirby–Bauer disc diffusion | NA | | Accession Number: 2037352060 |
| 184 | Dhivya K | 2024 | Tamil Nadu | Chennai | Hospital | Prospective cross-sectional | 2023 | Blood | NA | CLSI | | 10.4103/ajprhc.ajprhc_122_23 |
| 185 | Kumar DV | 2024 | New Delhi | New Delhi | Hospital | Prospective observational cross-sectional | 2020 | Blood | NA | NA | | 10.4038/sljch.v53i1.10702 |
| 186 | Saini S | 2024 | Haryana | Panipat | Hospital | Prospective cross-sectional | 2022 | Blood | NA | CLSI | | 10.48047/jcdr.2024.15.01.04 |
| 187 | Varghese G | 2024 | Uttar Pradesh | Lucknow | Laboratory | ?Prospective Observational | 2022 | Blood | Kirby–Bauer disc diffusion | CLSI | | 10.1016/j.diagmicrobio.2024.116354 |
| 188 | NCDC(NARS-Net)-Semi-Annual Report 2024 | 2024 | 60 state medical colleges in 27 states, & 6 UTs | NA | Laboratory | Prospective | 2024 | Blood | Disc diffusion | CLSI | | https://ncdc.mohfw.gov.in/reports/ |
