# Supplementary figures and images for: Burden of antimicrobial resistance in culture-confirmed Salmonella Typhi isolates in India from 1977 to 2024: A systematic review and meta-analysis
Source: PLoS Negl Trop Dis. 2026 Apr 16;20(4):e0014206. doi: 10.1371/journal.pntd.0014206 (PMC13108858; doi:10.1371/journal.pntd.0014206)

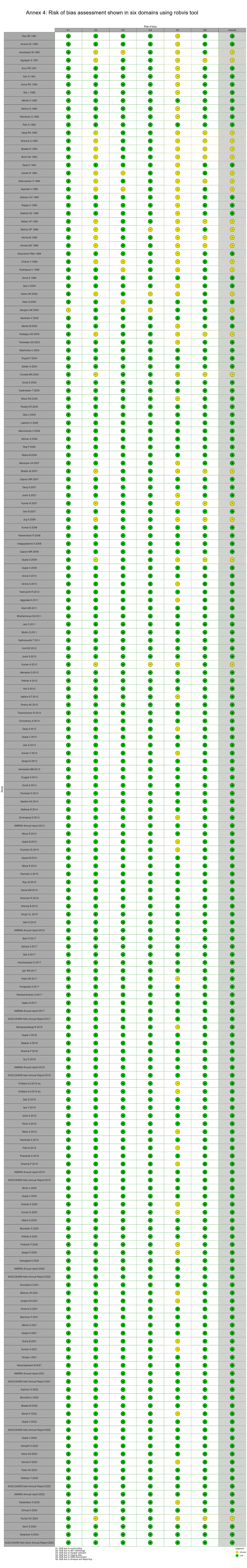

Supplement: S4 Annex — (TIF) [file pntd.0014206.s004.tif]

## Annex 9: The funnel plots of four anti-microbial resistance groups showing publication bias

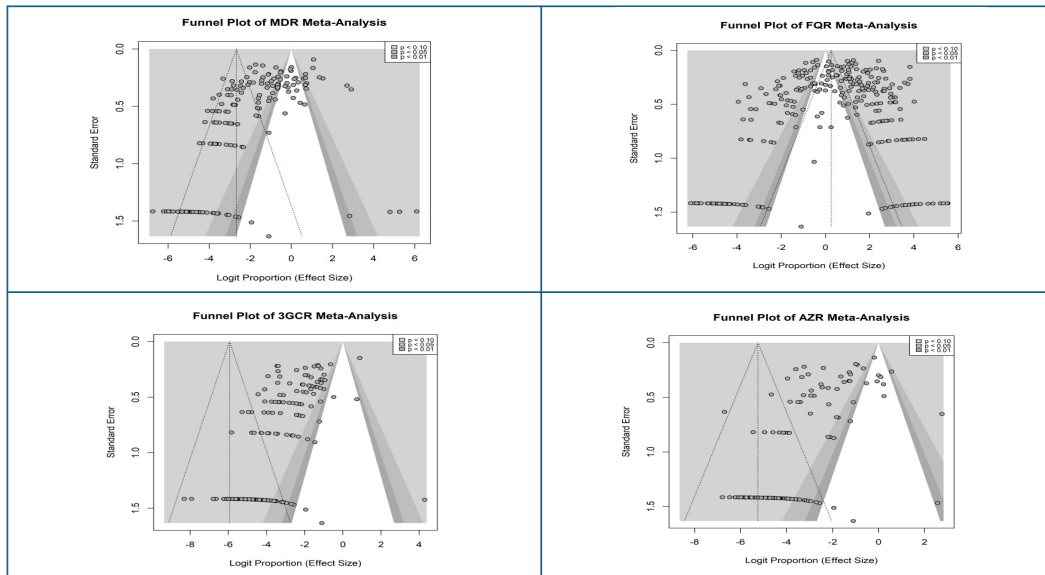

Supplement: S9 Annex — (PDF) [file pntd.0014206.s009.pdf]

Annex 10: Forest plot showing proportion of fluoroquinolone-resistant (FQR) S.Typhi in India since 2015.

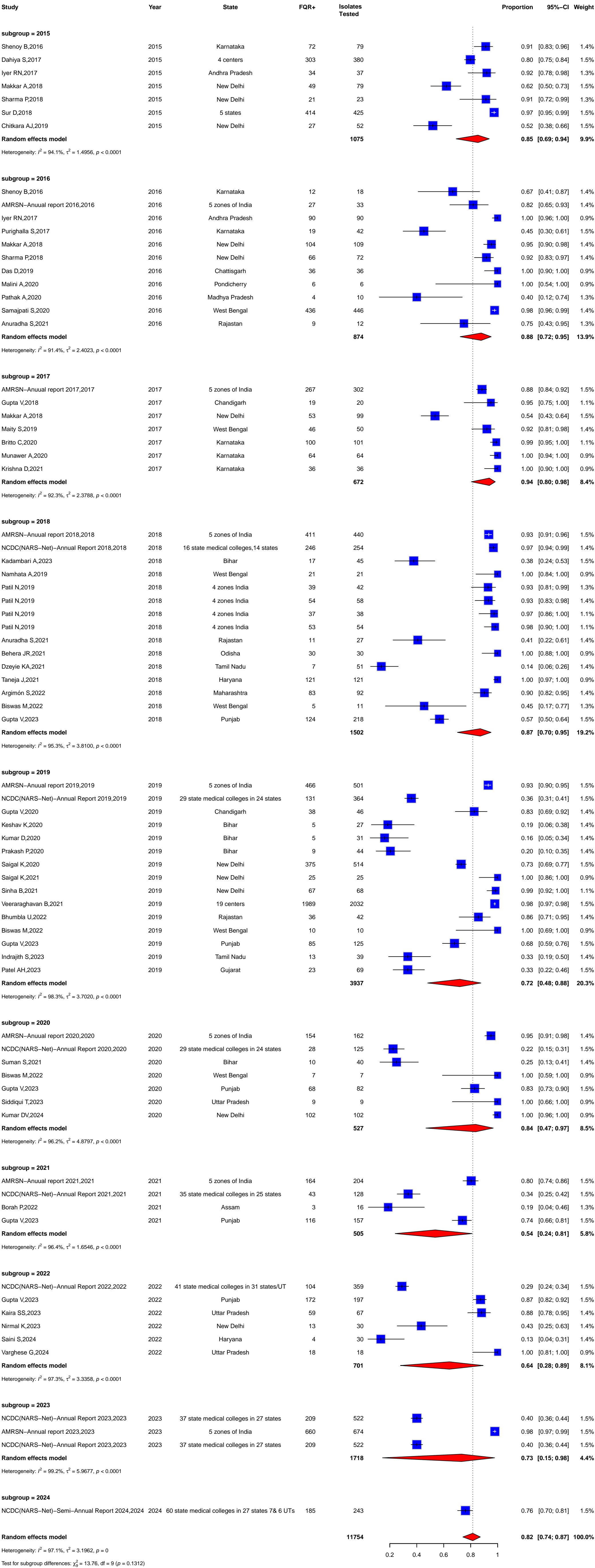

Supplement: S10 Annex — (PDF) [file pntd.0014206.s010.pdf]

Annex 11: Forest plot showing proportion of multi-drug resistant (MDR) S.Typhi in India since 2015

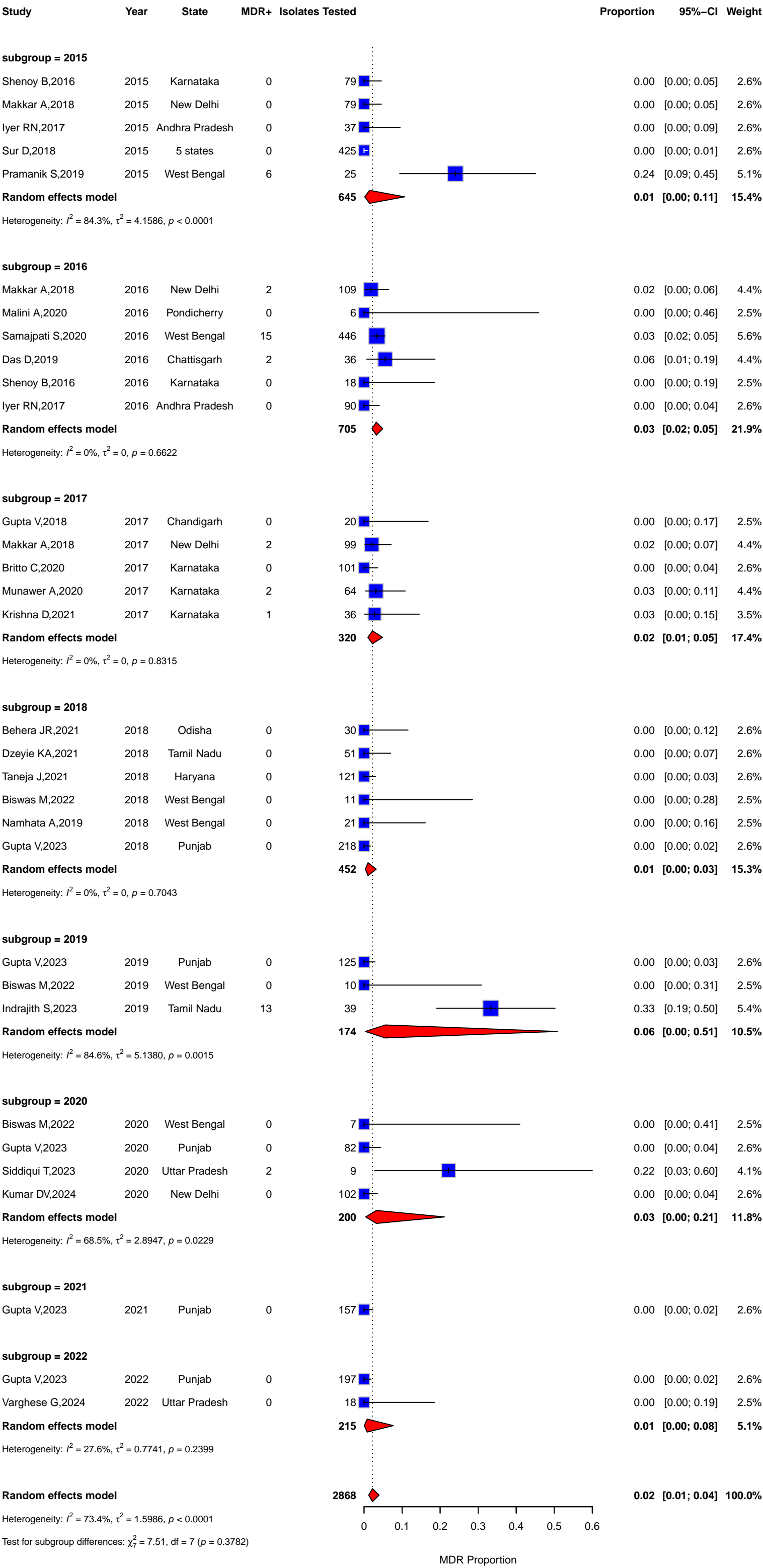

Supplement: S11 Annex — (PDF) [file pntd.0014206.s011.pdf]

Annex 13: Forest plot showing proportion of azithromycin-resistant (AZR) S.Typhi in India since 2015

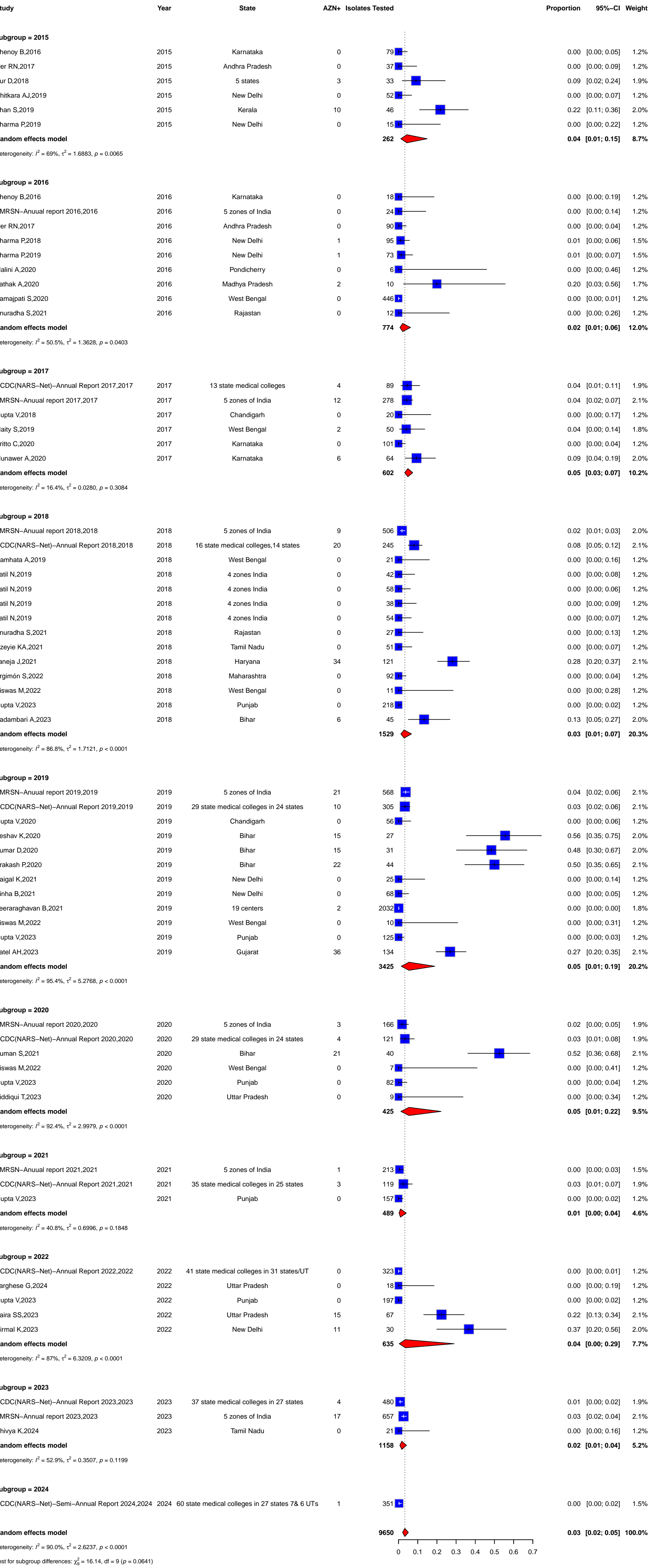

Supplement: S13 Annex — (PDF) [file pntd.0014206.s013.pdf]
