# Supplementary material for: Burden of antimicrobial resistance in culture-confirmed Salmonella Typhi isolates in India from 1977 to 2024: A systematic review and meta-analysis
Source: PLoS Negl Trop Dis. 2026 Apr 16;20(4):e0014206. doi: 10.1371/journal.pntd.0014206 (PMC13108858; doi:10.1371/journal.pntd.0014206)
Supplement: S5 Annex — (DOCX) [file pntd.0014206.s005.docx]

**Annex 5:** The characteristics of antimicrobial susceptibility tests (AST), source of samples used, study data points and studies that could not be geo-linked

**Characteristics of AST:** The disc diffusion was the most commonly reported AST method (n=106). Other methods mentioned included MIC (n=6), Vitek 2 (n=6), or combinations(n=35), while 35 studies did not specify the techniques used. The CLSI was the most commonly used standard guideline for interpreting AST results (n=96); others reported NCCLS (n=17), the British Society for Antimicrobial Chemotherapy (BSAC) (n=1), or combinations (n=3), and 71 papers did not specify the AST guidelines used.

**Sample sources of *S*. Typhi:** The most frequently reported source of *S.* Typhi isolates was blood, documented in 151 studies; the remainder reported multiple sources, including blood, bone marrow, urine, or cerebrospinal fluid.

**Study data points:** Of the 188 studies, 34 provided AST results over multiple years, which, when organised by year, yielded an additional 127 data points. Six multi-centre studies and 17 government reports did not specify the exact locations of the sample collection. Consequently, these studies were aggregated into a single data point for analysis and not assigned to any specific state in the state-wise analysis. Combining all this information, we had 315 study data points derived from 188 papers.

**Geo-linking:** We were unable to geo-link 10,886 isolates. Although the NCDC 2024 report included isolates from 60 medical colleges across 31 states and union territories, it did not provide state-wise data, making it impossible to allocate isolates to specific states. Similarly, one study collected AST samples from four states, and another from five; however, neither provided a breakdown of *S*. Typhi isolates by state. Altogether, six multi-centre studies and 17 government reports detailed sample collection from various Indian locations (e.g., 15 centres, 14 hospitals, four zones, six sites, 18 regions, 19 centres, 20 research centres, and 31 states) could not be allocated to specific states.
