# Supplementary material for: Burden of antimicrobial resistance in culture-confirmed Salmonella Typhi isolates in India from 1977 to 2024: A systematic review and meta-analysis
Source: PLoS Negl Trop Dis. 2026 Apr 16;20(4):e0014206. doi: 10.1371/journal.pntd.0014206 (PMC13108858; doi:10.1371/journal.pntd.0014206)

**Annex 6**: Bubble map of 29,921 *S.*Typhi isolates by Indian states identified in the systematic review. Darkness of colour and bubble size are proportional to sample size. (Base map: India administrative boundaries from Natural Earth (Admin 1 – States and Provinces; https://www.naturalearthdata.com), public domain, rendered using Everviz.)


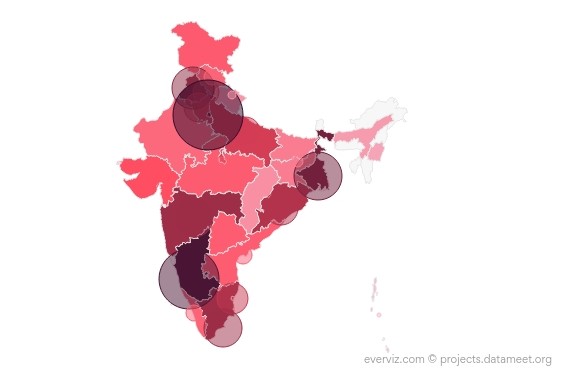

Supplement: S6 Annex — Darkness of colour and bubble size are proportional to sample size. (Base map: India administrative boundaries from Natural Earth (Admin 1 – States and Provinces; https://www.naturalearthdata.com), public domain, rendered using Everviz.). (DOCX) [file pntd.0014206.s006.docx]
