# Supplementary material for: Burden of antimicrobial resistance in culture-confirmed Salmonella Typhi isolates in India from 1977 to 2024: A systematic review and meta-analysis
Source: PLoS Negl Trop Dis. 2026 Apr 16;20(4):e0014206. doi: 10.1371/journal.pntd.0014206 (PMC13108858; doi:10.1371/journal.pntd.0014206)

Annex 7: Meta-analysis forest plot showing proportion of multi-drug resistant (MDR) S.Typhi in India by year

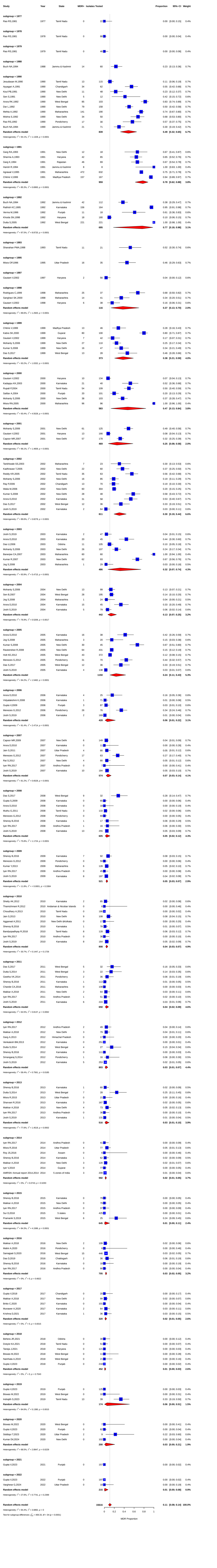

Supplement: S7 Annex — (PDF) [file pntd.0014206.s007.pdf]
