# Supplementary material for: Burden of antimicrobial resistance in culture-confirmed Salmonella Typhi isolates in India from 1977 to 2024: A systematic review and meta-analysis
Source: PLoS Negl Trop Dis. 2026 Apr 16;20(4):e0014206. doi: 10.1371/journal.pntd.0014206 (PMC13108858; doi:10.1371/journal.pntd.0014206)

Annex 8: Forest plot showing proportion of fluoroquinolone-resistant (FQR) S.Typhi in India by year

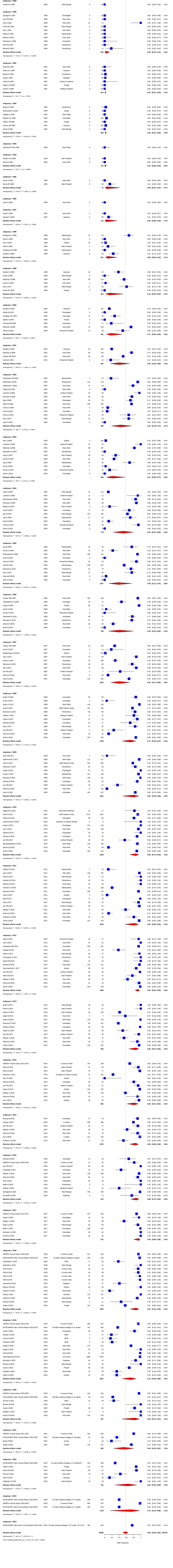

Supplement: S8 Annex — (PDF) [file pntd.0014206.s008.pdf]
