# Supplementary material for: Burden of antimicrobial resistance in culture-confirmed Salmonella Typhi isolates in India from 1977 to 2024: A systematic review and meta-analysis
Source: PLoS Negl Trop Dis. 2026 Apr 16;20(4):e0014206. doi: 10.1371/journal.pntd.0014206 (PMC13108858; doi:10.1371/journal.pntd.0014206)

Annex 12: Forest plot showing proportion of 3rd generation cephalosporins-resistant (3GCR) S.Typhi in India since 2015

Study	Year	State	3GCR+	Isolates Tested	Proportion	95%−CI	Weight
**subgroup = 2015**							
Shenoy B,2016	2015	Karnataka	0	79	0.00	[0.00; 0.05]	1.0%
Dahiya S,2017	2015	4 centers	0	380	0.00	[0.00; 0.01]	1.0%
Iyer RN,2017	2015	Andhra Pradesh	0	37	0.00	[0.00; 0.09]	1.0%
Sharma P,2018	2015	New Delhi	0	23	0.00	[0.00; 0.15]	1.0%
Chitkara AJ,2019	2015	New Delhi	0	52	0.00	[0.00; 0.07]	1.0%
Pramanik S,2019	2015	West Bengal	0	25	0.00	[0.00; 0.14]	1.0%
**Random effects model**				**596**	**0.01**	**[0.00; 0.03]**	**6.1%**
Heterogeneity: I^2 = 0%, τ^2 = 0, p = 0.7544							
**subgroup = 2016**							
Shenoy B,2016	2016	Karnataka	0	18	0.00	[0.00; 0.19]	1.0%
AMRSN−Anuual report 2016,2016	2016	5 zones of India	0	33	0.00	[0.00; 0.11]	1.0%
Iyer RN,2017	2016	Andhra Pradesh	0	90	0.00	[0.00; 0.04]	1.0%
Purighalla S,2017	2016	Karnataka	5	42	0.12	[0.04; 0.26]	2.0%
Ramachandran A,2017	2016	Tamil Nadu	2	68	0.03	[0.00; 0.10]	1.7%
Sharma P,2018	2016	New Delhi	0	72	0.00	[0.00; 0.05]	1.0%
Sharma P,2018	2016	New Delhi	0	95	0.00	[0.00; 0.04]	1.0%
Das D,2019	2016	Chattisgarh	0	36	0.00	[0.00; 0.10]	1.0%
Malini A,2020	2016	Pondicherry	0	6	0.00	[0.00; 0.46]	1.0%
Pathak A,2020	2016	Madhya Pradesh	2	10	0.20	[0.03; 0.56]	1.6%
Samajpati S,2020	2016	West Bengal	0	446	0.00	[0.00; 0.01]	1.0%
Anuradha S,2021	2016	Rajastan	0	12	0.00	[0.00; 0.26]	1.0%
**Random effects model**				**928**	**0.03**	**[0.01; 0.06]**	**14.5%**
Heterogeneity: I^2 = 55.8%, τ^2 = 1.4061, p = 0.0094							
**subgroup = 2017**							
AMRSN−Anuual report 2017,2017	2017	5 zones of India	5	334	0.01	[0.00; 0.03]	2.0%
Gupta V,2018	2017	Chandigarh	0	20	0.00	[0.00; 0.17]	1.0%
Maity S,2019	2017	West Bengal	3	50	0.06	[0.01; 0.17]	1.9%
Britto C,2020	2017	Karnataka	0	101	0.00	[0.00; 0.04]	1.0%
Munawer A,2020	2017	Karnataka	0	64	0.00	[0.00; 0.06]	1.0%
Krishna D,2021	2017	Karnataka	0	36	0.00	[0.00; 0.10]	1.0%
**Random effects model**				**605**	**0.02**	**[0.01; 0.05]**	**8.0%**
Heterogeneity: I^2 = 11.3%, τ^2 = 0.3100, p = 0.3432							
**subgroup = 2018**							
NCDC(NARS−Net)−Annual Report 2018,2018	2018	16 state medical colleges,14 states	16	202	0.08	[0.05; 0.13]	2.2%
Namhata A,2019	2018	West Bengal	0	21	0.00	[0.00; 0.16]	1.0%
Patil N,2019	2018	4 zones India	0	42	0.00	[0.00; 0.08]	1.0%
Patil N,2019	2018	0	0	58	0.00	[0.00; 0.06]	1.0%
Patil N,2019	2018	0	0	38	0.00	[0.00; 0.09]	1.0%
Patil N,2019	2018	0	0	54	0.00	[0.00; 0.07]	1.0%
Anuradha S,2021	2018	Rajastan	0	27	0.00	[0.00; 0.13]	1.0%
Behera JR,2021	2018	Odisha	0	30	0.00	[0.00; 0.12]	1.0%
Dzeyie KA,2021	2018	Tamil Nadu	7	51	0.14	[0.06; 0.26]	2.0%
Taneja J,2021	2018	Haryana	26	121	0.21	[0.15; 0.30]	2.2%
Argimón S,2022	2018	Maharashtra	12	92	0.13	[0.07; 0.22]	2.1%
Biswas M,2022	2018	West Bengal	0	11	0.00	[0.00; 0.28]	1.0%
Gupta V,2023	2018	Punjab	0	218	0.00	[0.00; 0.02]	1.0%
Kadambari A,2023	2018	Bihar	9	45	0.20	[0.10; 0.35]	2.1%
**Random effects model**				**1010**	**0.05**	**[0.02; 0.11]**	**19.8%**
Heterogeneity: I^2 = 70.2%, τ^2 = 1.3674, p < 0.0001							
**subgroup = 2019**							
AMRSN−Anuual report 2019,2019	2019	5 zones of India	14	448	0.03	[0.02; 0.05]	2.2%
NCDC(NARS−Net)−Annual Report 2019,2019	2019	29 state medical colleges in 24 states	2	301	0.01	[0.00; 0.02]	1.7%
Gupta V,2020	2019	Chandigarh	0	56	0.00	[0.00; 0.06]	1.0%
Keshav K,2020	2019	Bihar	7	27	0.26	[0.11; 0.46]	2.0%
Kumar D,2020	2019	Bihar	7	31	0.23	[0.10; 0.41]	2.0%
Prakash P,2020	2019	Bihar	11	44	0.25	[0.13; 0.40]	2.1%
Saigal K,2020	2019	New Delhi	1	514	0.00	[0.00; 0.01]	1.4%
Sinha B,2021	2019	New Delhi	0	68	0.00	[0.00; 0.05]	1.0%
Veeraraghavan B,2021	2019	19 centers	0	2032	0.00	[0.00; 0.00]	1.0%
Bhumbla U,2022	2019	Rajastan	3	42	0.07	[0.01; 0.19]	1.9%
Biswas M,2022	2019	West Bengal	1	10	0.10	[0.00; 0.45]	1.4%
Gupta V,2023	2019	Punjab	4	125	0.03	[0.01; 0.08]	1.9%
Indrajith S,2023	2019	Tamil Nadu	0	39	0.00	[0.00; 0.09]	1.0%
Patel AH,2023	2019	Gujarat	0	135	0.00	[0.00; 0.03]	1.0%
**Random effects model**				**3872**	**0.03**	**[0.01; 0.07]**	**21.7%**
Heterogeneity: I^2 = 87.6%, τ^2 = 3.5157, p < 0.0001							
**subgroup = 2020**							
AMRSN−Anuual report 2020,2020	2020	5 zones of India	1	158	0.01	[0.00; 0.03]	1.4%
NCDC(NARS−Net)−Annual Report 2020,2020	2020	29 state medical colleges in 24 states	3	123	0.02	[0.01; 0.07]	1.9%
Suman S,2021	2020	Bihar	11	40	0.28	[0.15; 0.44]	2.1%
Biswas M,2022	2020	West Bengal	0	7	0.00	[0.00; 0.41]	1.0%
Gupta V,2023	2020	Punjab	0	82	0.00	[0.00; 0.04]	1.0%
Siddiqui T,2023	2020	Uttar Pradesh	0	9	0.00	[0.00; 0.34]	1.0%
Kumar DV,2024	2020	New Delhi	12	102	0.12	[0.06; 0.20]	2.1%
**Random effects model**				**521**	**0.05**	**[0.01; 0.15]**	**10.5%**
Heterogeneity: I^2 = 80.9%, τ^2 = 1.8892, p < 0.0001							
**subgroup = 2021**							
AMRSN−Anuual report 2021,2021	2021	5 zones of India	3	212	0.01	[0.00; 0.04]	1.9%
NCDC(NARS−Net)−Annual Report 2021,2021	2021	35 state medical colleges in 25 states	2	125	0.02	[0.00; 0.06]	1.7%
Gupta V,2023	2021	Punjab	2	157	0.01	[0.00; 0.05]	1.7%
**Random effects model**				**494**	**0.01**	**[0.01; 0.03]**	**5.3%**
Heterogeneity: I^2 = 0%, τ^2 = 0, p = 0.9739							
**subgroup = 2022**							
Gupta V,2023	2022	Punjab	0	197	0.00	[0.00; 0.02]	1.0%
Kaira SS,2023	2022	Uttar Pradesh	7	67	0.10	[0.04; 0.20]	2.1%
Nirmal K,2023	2022	New Delhi	2	30	0.07	[0.01; 0.22]	1.7%
Saini S,2024	2022	Haryana	0	30	0.00	[0.00; 0.12]	1.0%
Varghese G,2024	2022	Uttar Pradesh	0	18	0.00	[0.00; 0.19]	1.0%
**Random effects model**				**342**	**0.04**	**[0.01; 0.12]**	**6.8%**
Heterogeneity: I^2 = 53.4%, τ^2 = 1.0583, p = 0.0721							
**subgroup = 2023**							
NCDC(NARS−Net)−Annual Report 2023,2023	2023	37 state medical colleges in 27 states	2	492	0.00	[0.00; 0.01]	1.7%
Dhivya K,2024	2023	Tamil Nadu	1	21	0.05	[0.00; 0.24]	1.4%
AMRSN−Annual report 2023,2023	2023	5 zones of India	21	661	0.03	[0.02; 0.05]	2.2%
**Random effects model**				**1174**	**0.02**	**[0.00; 0.07]**	**5.3%**
Heterogeneity: I^2 = 75.7%, τ^2 = 1.2099, p = 0.0164							
**subgroup = 2024**							
NCDC(NARS−Net)−Semi−Annual Report 2024,2024	2024	60 state medical colleges in 27 states 7& 6 UTs	4	385	0.01	[0.00; 0.03]	2.0%
**Random effects model**				**9927**	**0.03**	**[0.02; 0.04]**	**100.0%**
Heterogeneity: I^2 = 80.0%, τ^2 = 1.6895, p < 0.0001							
Test for subgroup differences: χ^2_9 = 13.76, df = 9 (p = 0.1310)							

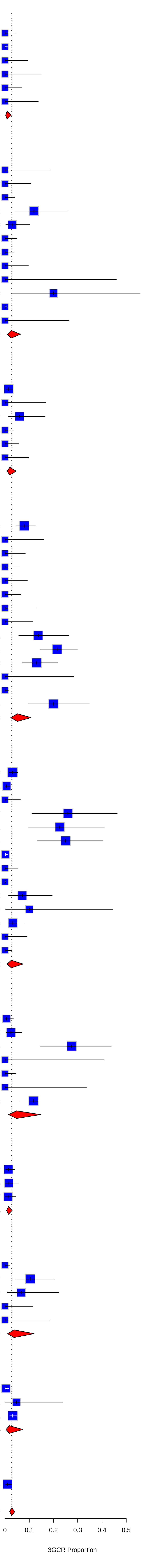

Supplement: S12 Annex — (PDF) [file pntd.0014206.s012.pdf]
