# Supplementary material for: Burden of antimicrobial resistance in culture-confirmed Salmonella Typhi isolates in India from 1977 to 2024: A systematic review and meta-analysis
Source: PLoS Negl Trop Dis. 2026 Apr 16;20(4):e0014206. doi: 10.1371/journal.pntd.0014206 (PMC13108858; doi:10.1371/journal.pntd.0014206)
Supplement: S14 Annex — (DOCX) [file pntd.0014206.s014.docx]

**Annex 14**: Prescription practices and their potential implications for AMR

Our findings indicate that the observed resistance patterns closely correlate with changing prescribing practices for typhoid treatment, both in India and globally, as treatment guidelines have evolved (5, 6). In the 1980s, clinicians commonly prescribed first-line antimicrobials such as ampicillin, chloramphenicol, and trimethoprim-sulfamethoxazole for typhoid fever. The MDR to these first-line antimicrobials peaked in the early 1990s, prompting a transition to fluoroquinolone prescriptions (7, 8). This change in prescription pattern matched the decline in MDR and the rise in FQR, which we inferred in our review. A longitudinal study tracking AMR to *S*. Typhi from 2000 to 2015 in three major hospitals in India showed a similar trend of declining MDR and increasing FQR (9). As FQR became more prevalent, third-generation cephalosporins gained popularity as a treatment option (7, 8) and we inferred a declining trend in FQR starting around 2020. However, it is essential to note that this decline could also be influenced by studies that do not categorise "intermediately resistant" or "intermediately susceptible" isolates as FQR. Nevertheless, we have no evidence to suggest that the reporting methods changed around 2020. Notably, former first-line treatment options, including ampicillin, chloramphenicol, and trimethoprim-sulfamethoxazole, are now rarely prescribed for typhoid fever in India. Consequently, sensitivity to these drugs is increasingly common, with almost zero MDR cases after 2020. Available evidence on typhoid fever treatment practices in India supports the theory that changing prescription patterns may be linked to the changes in AMR. A cross-sectional analysis of medical audit data on antimicrobial prescriptions for typhoid fever from 2013 to 2015 revealed a decrease in the proportion of quinolone prescriptions, alongside an increase in cephalosporin prescriptions (10). In 2013, quinolones (classified as J01M under the WHO Anatomical Therapeutic Chemical (ATC) classification system) accounted for 24.5% of prescriptions for typhoid fever. This percentage decreased to 22.4% in 2014 and further to 22.2% in 2015 (10). In contrast, the proportion of cephalosporin prescriptions (classified as J01D under the WHO ATC) for typhoid fever increased from 32.0% in 2013 to 34.1% in 2014, then slightly decreased to 32.8% in 2015. Additionally, 34% of all typhoid fever prescriptions consisted of combinations of multiple antimicrobials (classified as J01R under the WHO ATC). In comparison, the three first-line MDR-defining antimicrobials—ampicillin, chloramphenicol, and trimethoprim-sulfamethoxazole—accounted for only 1.5% of all typhoid fever prescriptions (10).

*S*. Typhi is known for its drug-adaptive nature, which develops or loses drug resistance in response to drug pressures consistent with changing antimicrobial consumption. Under drug pressure, S. Typhi acquires drug resistance through mobile genetic elements such as plasmids or composite transposons harbouring AMR genes, or through mutations in its chromosomal genes (11). When drug pressure is taken out, *S*. Typhi become drug sensitive again, which is clearly demonstrated for MDR, while not so clear for FQR (12). Some laboratory-based studies have shown that the acquisition of drug resistance through mutations incurs "fitness costs," impairing specific cellular processes, which can lead to a loss of drug resistance in a drug-free environment (13), while other studies have reported limited fitness costs for single mutations mediating fluoroquinolone resistance (14). There is also laboratory evidence suggesting that fluoroquinolone-resistant *S*. Typhi may lose its virulence, potentially decreasing its transmission and overall prevalence over time, but its significance in population settings is not known (15).

**References**

1. CLSI. Clinical and Laboratory Standards Institute Performance Standards for Antimicrobial Susceptibility Testing; 25th Informational Supplement. CLSI Document M100-S25, Clinical and Laboratory Standards Institute, Wayne, PA. 2015.

2. Britto CD, John J, Verghese VP, Pollard AJ. A systematic review of antimicrobial resistance of typhoidal *Salmonella* in India. Indian J Med Res. 2019;149(2):151-63.

3. Hayden JA, van der Windt DA, Cartwright JL, Côté P, Bombardier C. Assessing bias in studies of prognostic factors. Ann Intern Med. 2013;158(4):280-6.

4. Higgins JPT, Morgan RL, Rooney AA, Taylor KW, Thayer KA, Silva RA, et al. A tool to assess risk of bias in non-randomized follow-up studies of exposure effects (ROBINS-E). Environ Int. 2024;186:108602.

5. World Health Organization. Typhoid vaccines: WHO position paper, March 2018 - Recommendations. Vaccine. 2019;37(2):214-6.

6. WHO. Model List of Essential Medicines - 23rd list 2023 [cited 2025 April 5,]. Available from: https://list.essentialmeds.org/?indication=219; https://www.who.int/groups/expert-committee-on-selection-and-use-of-essential-medicines/essential-medicines-lists.

7. Hamdulay K, Rawekar R, Tayade A, Kumar S, Acharya S. Evolving Epidemiology and Antibiotic Resistance in Enteric Fever: A Comprehensive Review. Cureus. 2024;16(6):e63070.

8. Dyson ZA, Klemm EJ, Palmer S, Dougan G. Antibiotic Resistance and Typhoid. Clin Infect Dis. 2019;68(Suppl 2):S165-S70.

9. Balaji V, Kapil A, Shastri J, Pragasam AK, Gole G, Choudhari S, et al. Longitudinal Typhoid Fever Trends in India from 2000 to 2015. Am J Trop Med Hyg. 2018;99(3_Suppl):34-40.

10. Fazaludeen Koya S, Hasan Farooqui H, Mehta A, Selvaraj S, Galea S. Quantifying antibiotic use in typhoid fever in India: a cross-sectional analysis of private sector medical audit data, 2013-2015. BMJ Open. 2022;12(10):e062401.

11. Chowdhury AR, Mukherjee D, Chatterjee R, Chakravortty D. Defying the odds: Determinants of the antimicrobial response of Salmonella Typhi and their interplay. Mol Microbiol. 2024;121(2):213-29.

12. Tanmoy AM, Hooda Y, Sajib MSI, Rahman H, Sarkar A, Das D, et al. Trends in antimicrobial resistance amongst Salmonella Typhi in Bangladesh: A 24-year retrospective observational study (1999-2022). PLoS Negl Trop Dis. 2024;18(10):e0012558.

13. Dunai A, Spohn R, Farkas Z, Lazar V, Gyorkei A, Apjok G, et al. Rapid decline of bacterial drug-resistance in an antibiotic-free environment through phenotypic reversion. Elife. 2019;8.

14. Baker S, Duy PT, Nga TV, Dung TT, Phat VV, Chau TT, et al. Fitness benefits in fluoroquinolone-resistant Salmonella Typhi in the absence of antimicrobial pressure. Elife. 2013;2:e01229.

15. Balleste-Delpierre C, Fabrega A, Ferrer-Navarro M, Mathur R, Ghosh S, Vila J. Attenuation of in vitro host-pathogen interactions in quinolone-resistant Salmonella Typhi mutants. J Antimicrob Chemother. 2016;71(1):111-22.
